# Effect of Polymer Structure on the Thermodynamics of Polyelectrolyte Complex Micelle Formation

**DOI:** 10.1021/acs.macromol.5c03639

**Published:** 2026-03-07

**Authors:** Vishnu L. Dharmaraj, Yun Fang, Matthew V. Tirrell

**Affiliations:** Pritzker School of Molecular Engineering, University of Chicago, Chicago, Illinois 60637, United States; Biological Sciences Division, Department of Medicine, University of Chicago, Chicago, Illinois 60637, United States; Pritzker School of Molecular Engineering, University of Chicago, Chicago, Illinois 60637, United States

## Abstract

Polyelectrolyte complex micelles (PCMs) are nanoparticles that form through the associative phase separation between hydrophilic neutral–charged block copolymers and oppositely charged polyelectrolytes, resulting in a dense, charged core surrounded by a stabilizing neutral corona. Among other applications, these constructs have been studied as nonviral vectors for therapeutic nucleic acid delivery for a variety of potential clinical indications. Although prior research has focused on tailoring PCM morphology and size by modifying block polyelectrolyte characteristics, thermodynamic considerations have received comparatively little attention in the design of PCMs. In this study, we explore the dependencies of PCM complexation thermodynamics, particularly the entropy of complexation, on polyelectrolyte block length and PCM structure. We employ scattering (DLS, SAXS, MALS) to characterize PCM structure, while using isothermal titration calorimetry to provide quantitative thermodynamic data. Compared to complexation between homopolymers, we observe that PCM formation involving block polyelectrolytes introduces an entropic cost related to the neutral corona-forming block. This penalty depends on the sizes of the charged blocks but is relatively insensitive to the neutral block size. Scattering results show that PCM complexation entropy is not correlated with indicators of corona chain conformation, such as brush height and corona surface chain density. Rather, for PCMs composed of polymers with equal charged block lengths, complexation entropy is correlated with monomer density within the core and corona. Our findings also suggest a negligible free polymer concentration in PCM formulations with net neutral charge. These insights advance the rational design of block copolymers for encapsulating a wide array of therapeutically relevant cargos and deepen our understanding of the factors governing PCM formation.

## Graphical Abstract

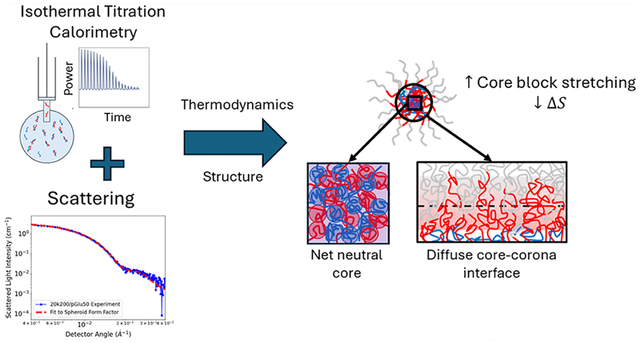

## INTRODUCTION

**Figure 1.**
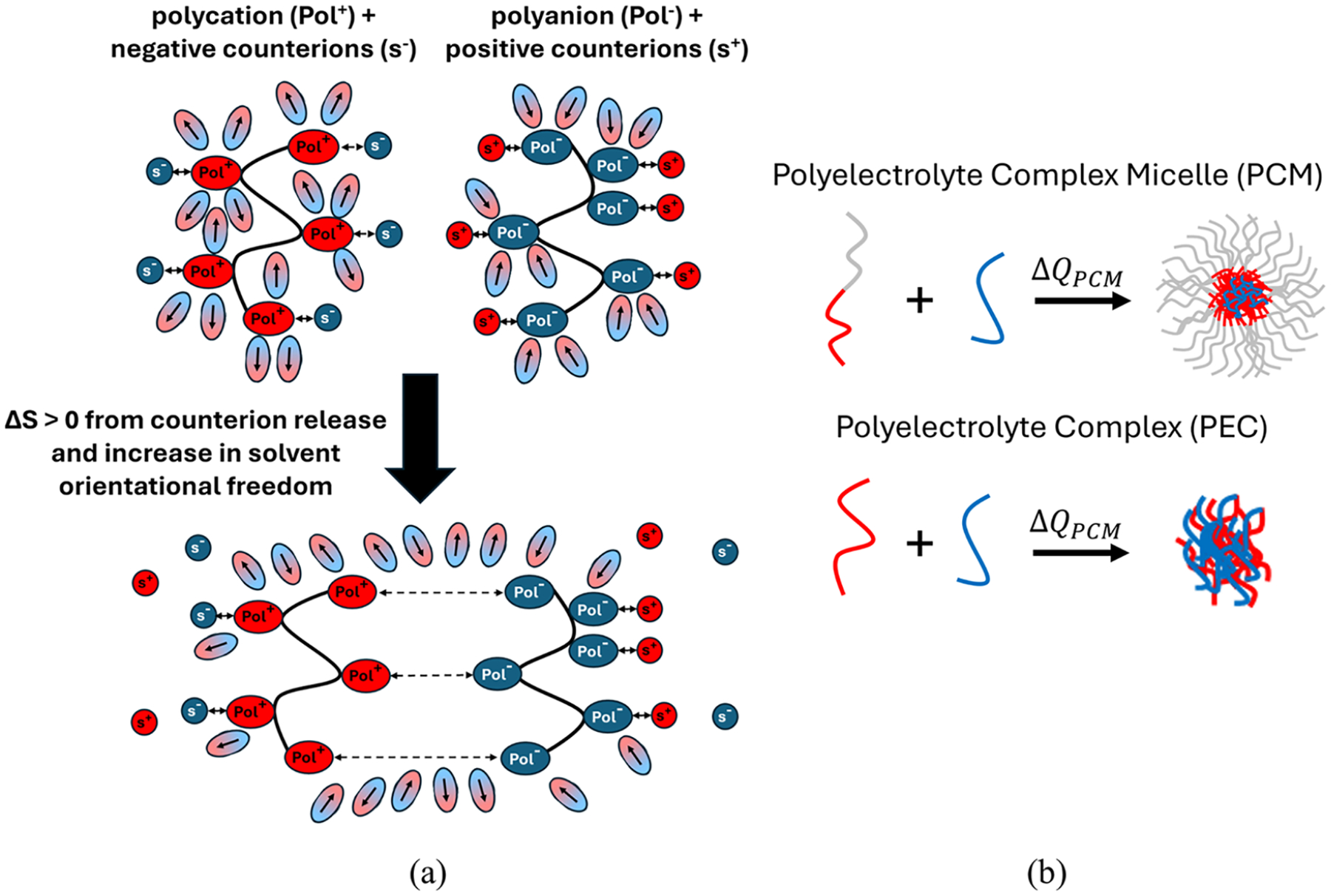
(a) Schematic of polyelectrolyte complexation where oppositely charged monomers form ion pairs with each other, releasing their counterions and increasing the entropy of complexation. An increase in solvent orientational entropy, as shown by the decreased alignment of solvent dipoles around the polyelectrolytes, also contributes to complexation, and is dictated by the solvent dielectric constant.^1^ (b) Schematic of polyelectrolyte complex micelles (PCMs) forming when a neutral block is added to the polycation as compared with polyelectrolyte complex (PEC) coacervates, where two homopolyelectrolytes combine in solution. The goal of this study is to understand the difference between the thermodynamic driving forces in these two systems.

Polyelectrolyte complex (PEC) coacervation is a liquid–liquid phase separation process driven by the associative interaction of oppositely charged (polycationic/polyanionic) macromolecules in aqueous solution, as well as counterion release and solvent rearrangement^1^ (Figure 1a). This phenomenon leads to the formation of a dense, polymer-rich coacervate phase in equilibrium with a dilute supernatant. Complex coacervation has garnered significant interest due to its relevance in biology and numerous applications.^2–7^ One application of polyelectrolyte complexes has been their use as drug delivery agents for a variety of charged, therapeutically relevant macromolecules, such as oligonucleotides and proteins.^8–11^ For successful delivery, cargos must be sequestered in nanoparticle complexes that remain in solution without aggregation or macrophase separation. To accomplish this with polyelectrolytes, a neutral, hydrophilic block of sufficient length is attached to either the polycation or polyanion. While the associative interactions between the polyelectrolytes still lead to the formation of a densely charged complex core, the neutral block forms a corona around it, leading to the formation of nanoparticles called polyelectrolyte complex micelles (PCMs) as shown in Figure 1b.

There are many variables (processing conditions, polymer structure/chemistry, etc.) that can affect the polyelectrolyte complexation process and the resulting material properties.^12–17^ In the context of PCMs and drug delivery, it is important to design polymers that have optimal interactions with a therapeutic cargo, such that PCMs readily form and are stable. Understanding the thermodynamic driving forces of complexation for PCMs and their dependencies is at the heart of this design process. An a priori expectation is that the entropy gain for PCM formation will be less than that for PEC formation, when all polyelectrolyte block lengths are equal, due to the entropy loss of confining and tethering the neutral chain in the corona.

**Figure 2.**
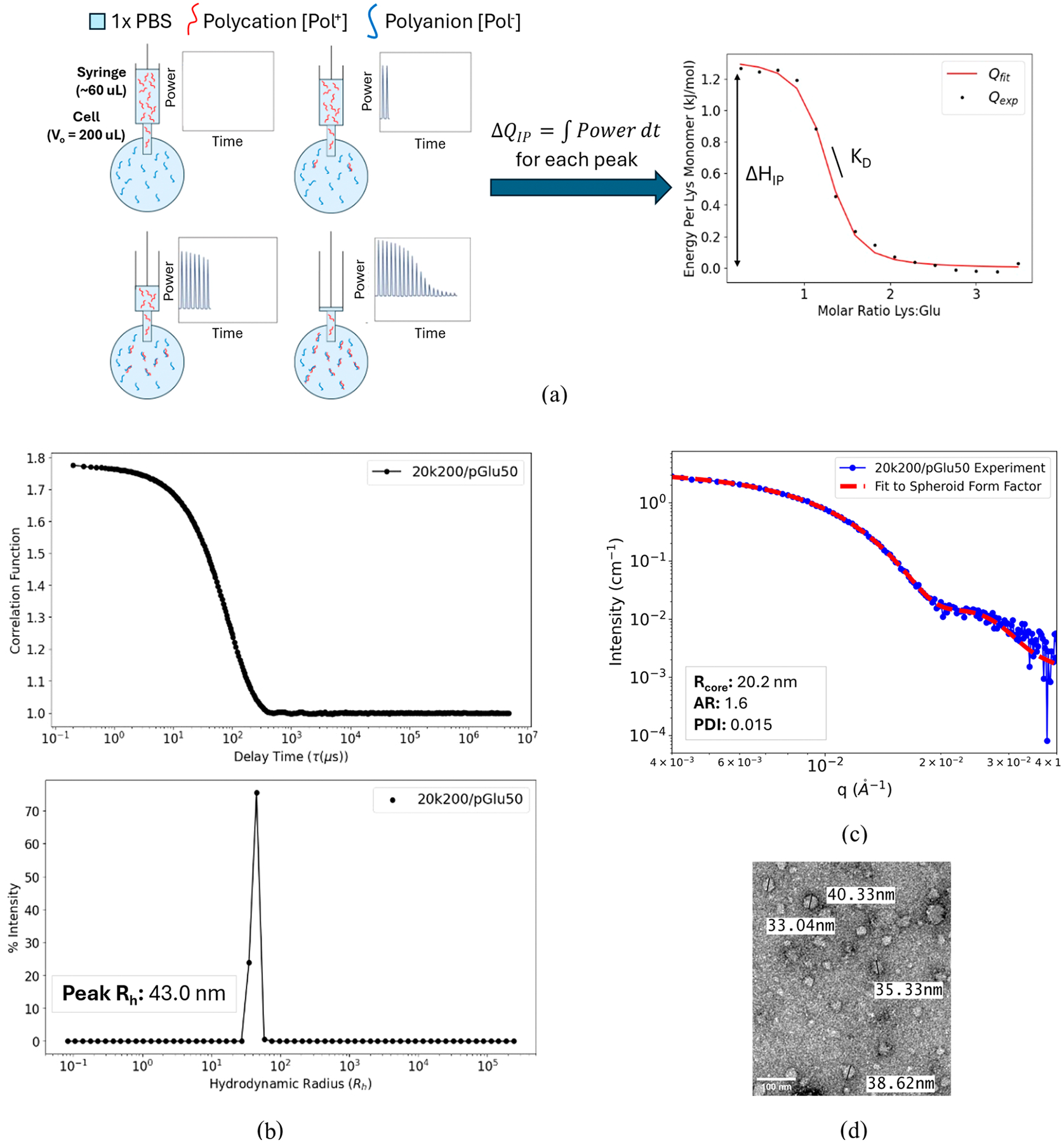
(a) Schematic of isothermal titration calorimetry (ITC) setup and sample ITC heat curve where data are fit to the ion pairing (IP) model. The ITC instrument monitors the change in heat, 𝚫*Q*, for the *i*^th^ injection as the polycation is injected and saturates the polyanion within the cell. Based on the ion pairing (IP) model, the height of the curve correlates with the enthalpy of ion pairing, 𝚫*H*_IP_, and the slope of the curve at the inflection point gives the dissociation constant, *K*_D_. (b) Sample dynamic light scattering (DLS) correlation curve and radial distribution of PCMs formed from combining PEG-(20 kDa)-pLys(200) and pGlu(50) (20k200/pGlu50 PCMs). Hydrodynamic radius (*R*_h_) distributions for PCMs are monodisperse and can be utilized to estimate the corona thickness (*h* = *R*_h_ – *R*_core_). (c) Sample small-angle X-ray scattering (SAXS) curve of 20k200/pGlu50 PCM core fit to a spheroidal form factor, where core radius (*R*_core_), aspect ratio (AR), and polydispersity index (PDI) are fitting parameters. (d) Sample transmission electron microscope (TEM) image of 20k200/pGlu50 PCMs. Labels indicate core diameters in the image, which are in line with the average *R*_core_ shown in Figure 2c. TEM (and SAXS) only shows PCM cores as the contrast in electron densities between the PEG corona and water is not large enough.

Experimental studies have utilized isothermal titration calorimetry (ITC), wherein one solution is titrated into the other, and the resulting heat output from each titration is monitored (Figure 2a).^18–21^ For our study, the polycation solution in the syringe is injected into a polyanion solution within a temperature regulated sample cell. Relevant thermodynamic parameters between the two molecules of interest, such as the Gibbs free energy, enthalpy and entropy changes, dissociation constant, and stoichiometry of binding can be extracted using appropriate models fit to the heat curve generated from the titration.

In previous studies,^19,20,22^ the heat from polyelectrolyte complexation (𝚫*Q*) has been modeled as the sum of enthalpies from two processes: ion pairing (𝚫*Q*_IP_) and complex coacervation (𝚫*Q*_c_). In the ion pairing (IP) model for polyelectrolytes, complexation is a reversible binding event wherein oppositely charged monomers form an ion pair, releasing their respective counterions in the process (eq 1, Figure 2a).


(1) $${\text{Pol}}^{+}{\text{s}}^{-}+{\text{Pol}}^{-}{\text{s}}^{+}↔{\text{Pol}}^{+}{\text{Pol}}^{-}+{\text{s}}^{-}{\text{s}}^{+}$$

(2) $$Q_{\text{IP}}(i)=n_{\text{IP}}𝚫H_{\text{IP}}\theta (i){\left[{\text{Pol}}^{-}\right]}_{\text{tot}}V_{0}$$

(3) $$K_{\text{D}}={\text{e}}^{𝚫G_{\text{IP}}/\text{RT}}=\frac{\left[{\text{Pol}}^{-}\right]\left[{\text{Pol}}^{+}\right]}{\left[{\text{Pol}}^{-}\cdot {\text{Pol}}^{+}\right]}=\frac{\left(1-\theta_{\text{IP}}\right)\left[{\text{Pol}}^{+}\right]}{\theta_{\text{IP}}}$$

The heat absorbed or released due to IP at the *i*^th^ injection is described in eq 2, where *V*_0_ is the volume of the cell, *n* is the stoichiometry of binding, [Pol^−^]_tot_ is the polyanion concentration within the cell at the start of the titration, and *θ*(*i*) is the fraction of polyanion sites that are bound in IPs after the *i*^th^ injection. *θ* is defined based on the concentrations of each charged species at the *i*^th^ injection, as well as the dissociation constant (*K*_D_) of ion pairing (eq 3). The normalized differential heat expelled, 𝚫*Q*_IP_ (*i*), for each injection is calculated by subtracting *Q*_IP_ (*i* – 1) from *Q*_IP_ (*i*) and normalizing by the number of moles of polycation injected, making a small correction for the volume of solution expelled from the cell at each injection. 𝚫*Q*_IP_ (*i*) is the quantity recorded by the ITC instrument at each injection in the titration. By fitting 𝚫*H*_IP_, *n*, and *K*_D_, the enthalpic and entropic contributions to ion pairing can be easily determined. Depending on the extent of coacervate formation (phase separation) at each point in the titration, which can be determined through other experiments such as turbidity, a separate set of analogous parameters can be used to fit the coacervation heat contribution (𝚫*Q*_c_).^19,20^ This framework has been used to describe homopolyelectrolyte complexation under a variety of conditions (salt concentration, pH, monomer/counterion species, polymer concentration, etc.).

The goal of this study is to apply this model to describe the thermodynamics of polyelectrolyte complexation within the context of PCM formation, by examining the effect of conjugating a neutral block to the polycation (Figure 1b). To the best of the authors’ knowledge, this is the first study that systematically examines PCM formation thermodynamics and its structural underpinnings. We utilize poly(ethylene glycol)-poly(L-lysine hydrochloride) (PEG-pLys) as the neutral-cationic block copolymer and poly(D,L-glutamic acid) sodium salt (pGlu) as the polyanion. In therapeutic applications, the polyanions are typically nucleic acids; the thermodynamics of their complexation to form PCMs is currently being studied in our laboratory. For now, PCMs composed of anionic polypeptides are the subject of this first study owing to (a) the database we have on these homopolymers,^20^ and (b) the simpler polymer physics of polypeptides.

We refer to a block copolymer (BCP) containing a PEG block with molecular weight (MW) of *m* kDa and a pLys block of *n* Lys units as a *m*k*n* BCP. For matched charged block length complexation, the pGlu homopolymer (HP) is not explicitly labeled since it is implied that the charged block lengths are the same. We systematically vary the lengths of the charged (pLys, pGlu) and neutral blocks (PEG) to examine their effect on the enthalpic and entropic contributions to complexation. Comparisons are also made to homopolyelectrolyte PEC analogues without a neutral block. To perform this comparison, experiments are performed in a background solution of phosphate-buffered saline (PBS, pH 7.4), which greatly reduces 𝚫*Q*_c_, eliminating the difference due to the macrophase separation in PECs. Performing experiments in PBS also: (1) simulates physiological pH and salt concentration under which PCMs would be used as drug delivery agents in the body, and (2) reduces kinetic effects of complexation due to charge screening, allowing for the sampling of different conformations to occur without hindrances due to a buildup of charge–charge interactions.

To better understand the physical origins for thermodynamic trends in PCM formation, we perform dynamic light scattering (DLS), small-angle X-ray scattering (SAXS), multi-angle light scattering (MALS), protein quantification using a CBQCA (3-(4-carboxybenzoyl)quinoline-2-carboxaldehyde) agent, and transmission electron microscopy (TEM) measurements. Specifically, SAXS is used to measure the size and shape of the PCM core and to measure PCM aggregation number (number of polymers in each micelle). Transmission electron microscopy (TEM) is used to confirm core shape and size, while MALS is used to confirm PCM molecular weight and aggregation number. CBQCA protein quantification is used to assess free polymer concentration PCM formulations. Lastly, DLS is used to measure the hydrodynamic radius (*R*_h_) of the entire PCM, thereby giving additional insight into the dimensions of the corona. Together, these techniques provide a comprehensive picture of PCM morphology.

Understanding the interplay between component polymer structure, overall PCM structure, and the thermodynamic factors involved in PCM formation, can be crucial to developing tailored polymers for the successful sequestration of a variety of molecules. In the context of drug delivery, this understanding can inform the rational design of stable BCP PCMs for the delivery of a variety of oligonucleotides and proteins with different sizes and secondary structures.

## EXPERIMENTAL METHODS

### Material Preparation

Poly(ethylene glycol)-poly(L-lysine hydrochloride) (PEG-pLys), poly(D,L-glutamic acid) sodium salt (pGlu), and poly(L-lysine hydrochloride) (pLys) were purchased from Alamanda Polymers. Molecular weights of all polymers and degrees of polymerization were provided by the manufacturer, and all polymers were used without further purification. These quantities, along with the chemical structure of the polymers used, are available in Table S1 and Figure S1, respectively. Stock solutions in Milli-Q water were prepared at a concentration of 500 *μ*M for each polymer. Solutions were vortexed for 1 min and sonicated for 10 min to allow for adequate solubilization. Stock solutions were stored in −20 °C in between experiments. When thawing back to room temperature, stock solutions were once again vortexed and sonicated prior to use. For each experiment, stock solutions were diluted using Milli-Q water. PBS was then added until polymer solutions contained a working concentration of 1× PBS. Solutions were vortexed for 3 min once PBS was added and allowed to sit for an additional 10 min. For ITC experiments, polymer component solutions were loaded separately. For other experiments, both polymer component solutions containing PBS were combined and allowed to sit for 15 min prior to measurement.

### Isothermal Titration Calorimetry (ITC)

ITC experiments were performed on a Malvern PEAQ ITC instrument. Charged monomer concentrations (polymer concentration × charged block length) were kept at approximately 500 *μ*M for the polyanion and 5 mM for the polycation (small differences are due to differences in DP of polymers as shown in Table S1). Adjustments were made to polyelectrolyte concentration depending on the signal-to-noise ratio of the heat curves generated. A table of charged monomer concentrations utilized for each sample combination can be found in Table S2.

Figure 2a shows a schematic of the ITC experimental setup. PEG-pLys or pLys (polycation) solution was injected in 18 increments of 2 *μ*L (extra solution was in the syringe to prevent the introduction of air bubbles) into a 200 *μ*L well of pGlu (polyanion) solution. At least 3 min were allowed for equilibration in between each injection. The power supplied by the ITC instrument to maintain a uniform temperature in the sample cell was monitored over time, and was integrated over time for each peak to yield the heat associated with each injection. In addition, for a particular polycation/polyanion combination, control data were collected for the titration of polycation into background solution (1× PBS), the titration of background solution into polyanion, and the titration of background solution into background solution without any other components. The heats measured from these blank tests were subtracted from the heat generated from the titration of the two polymer components at each injection to correct for entropic contributions due to volume changes. In addition, the integrated heats at the end of the titration were calibrated to zero heat to eliminate any systematic error associated with the instrument. While the control heats were negligible, this ensured that the final adjusted heat curves corresponded to only the complexation interactions between the two charged polymeric components in the titration. Heat curves were fit to the ion pairing (IP) model outlined previously (Figure 2a). Measurements were performed at least in triplicate. Statistical comparisons between homopolymer–homopolymer (HP–HP) and block copolymer–homopolymer (BCP–HP) complexation containing equivalent charged block lengths were performed using ANOVA tests for multiple means. A full list of fitting parameters for the ITC model for all combinations tested can be found in Table S4. A full table of p-values for comparisons can be found in Figure S2.

### Dynamic Light Scattering (DLS)

DLS measurements were performed using a Wyatt Möbius dynamic light scattering instrument. The instrument was equilibrated at 25 °C, and measurements were conducted in quartz crystal cuvettes with a sample volume of 50 *μ*L. A table of the concentrations utilized for each sample combination can be found in Table S3. Unless otherwise stated, concentrations in Table S3 were utilized for subsequent techniques (TEM, SAXS). Data acquisition was carried out using the Wyatt Möbius software, which measured signal at a 165° scattering angle for a duration of 5 s for each of 10 measurements. The autocorrelation function was analyzed using regularization methods to determine the hydrodynamic radius (*R*_h_) and polydispersity index (PDI) from the Stokes–Einstein relationship. A sample DLS correlation function and *R*_h_ distribution are shown in Figure 2b, with tabulated averaged results shown in Table S5.

### Small-Angle X-ray Scattering (SAXS)

SAXS experiments were performed in a similar manner to those outlined previously.^23–25^ Further discussion of the PCM assembly technique and SAXS fitting can be found in these works, but the overall procedure will be summarized here. Measurements were made at beamline 12–ID–B of the Advanced Photon Source at Argonne National Laboratory. Glycerol was added to all samples for a final concentration of 1% by volume to minimize radiation damage. Micelle samples were irradiated in a thin-walled quartz capillary flow cell with a photon energy of 14 keV. Forty-five consecutive 0.2 s exposures were collected and screened for evidence of radiation damage. During data collection, solutions were flowed through the cell to maximize the exposed volume and reduce the radiation dose per unit volume. Data were reduced and averaged in MATLAB at the beamline. Background subtraction and model fitting were performed using the modeling macros distributed with the Irena software package^26^ for Igor Pro. We use a Schulz-Zimm distribution and a rigid spheroid shape factor when modeling SAXS data to fit the PCM core radius, aspect ratio, and particle size polydispersity (Table S5). While these are core–shell nanoparticles, the PEG corona has a very similar electron density to water. As a result, essentially all of the scattering comes from the charged core. The corona is almost transparent in this technique, meaning that its thickness can only be assessed through other techniques, such as DLS. PCM samples were prepared in the same manner as for ITC, with component polyelectrolyte stock solutions being prepared separately in PBS. To ensure good signal, the charged monomer concentration was adjusted as necessary, with final charged monomer concentrations being listed in Table S3. Forward scattering intensity, *I*(*q* = 0), was extrapolated from a linear fit of a Guinier plot (ln(*I*) vs *q*^2^) in the region where 0.5 < *qR*_*g*_ < 1.3. A sample SAXS curve along with the corresponding fitting is shown in Figure 2c.

### Multi-Angle Light Scattering (MALS)

Molecular weights of formulated PCMs were measured in batch mode using a Wyatt DAWN HELEOS-II multi-angle light scattering detector equipped with a Wyatt Optilab T-rEX differential refractive index detector. PCM solutions were formulated at a combined polymer concentration of 0.2, 0.16, 0.12, 0.08, and 0.04 mg/mL in PBS. Samples were filtered (0.22 *μ*m) and injected into the flow cell. Refractive index (RI) and light scattering data were simultaneously measured by both instruments in series. Data were analyzed using ASTRA software (Wyatt Technology), and molecular weights were calculated using the Zimm model by extrapolating scattering data to both zero-angle and zero-concentration. A d*n*/d*c* value of 0.17 mL/g was measured for PEG-pLys samples in PBS and was utilized in the previously mentioned Zimm analysis. Assuming charge neutrality of the PCMs, PCM molecular weights were then translated to micelle BCP aggregation numbers (*n*_BCP_), which are recorded in Table S6 for select formulations.

### CBQCA Protein Quantitation

To assess the amount of free polymer not in PCMs within a given formulation, PCM solutions were run through a CBQCA Protein Quantitation assay (Thermo Fisher Scientific). PCM solutions were prepared at 0.5 mg/mL total polymer concentration in PBS. To separate unbound, free polymers from the micelle, the solution was separated into filtrate and retentate fractions by a 50 kDa cutoff membrane (Amicon Ultra-0.5 centrifugal filter devices, Millipore). DLS measurements were taken of both the filtrate and retentate to ensure micelles were only present in the latter. Both solutions were then run through the CBQCA assay according to the manufacturer’s protocol with minor modifications. Since the CBQCA agent is used to track the presence of primary amine groups, signal primarily comes from the pLys groups in the BCP. Briefly, samples (filtrate and retentate) and standards (PEG-pLys solutions at the concentration required to make the 0.5 mg/mL solution followed by 4, 2× serial dilutions) were prepared in black 96-well plates to minimize background fluorescence. Each well received 135 *μ*L of sample or standard, followed by 10 *μ*L of 2 mM CBQCA reagent, and 5 *μ*L of 20 mM mandelonitrile. The reaction volume also contained 0.05 M BuPH borate buffer. Plates were incubated and shaken at room temperature in the dark for 2 h. Fluorescence, a proxy for the BCP concentration in each solution, was measured using a plate reader (excitation/emission: 465/550 nm). Final protein concentrations were determined from the standard curve generated under identical conditions and the mass of protein in the filtrate and retentate were calculated. This yielded the percentage of free BCP in each solution, which is reported for each tested formulation in Table S7.

### Transmission Electron Microscopy (TEM)

PCM samples were imaged using an FEI Tecnai Spirit BioTWIN transmission electron microscope operated at an accelerating voltage of 120 kV with a LaB_6_ filament source. Samples were prepared by depositing a small aliquot (typically 5 *μ*L) onto a carbon-coated copper grid, followed by blotting and air-drying. Grids were stained with 1–2% (w/v) uranyl acetate for contrast enhancement. Images were acquired using a bottom-mounted CCD camera and processed using the manufacturer’s software or ImageJ. A sample TEM image is found in Figure 2d.

## RESULTS AND DISCUSSION

To extract relevant thermodynamic quantities from integrated ITC heat curves, we utilize the ion pairing (IP) model for coacervation described previously and in other studies.^20^ A background solution of 1× PBS (ionic strength of ≈ 160 mM salt) was utilized to reduce any kinetic barriers to PCM formation and to reduce the heat contribution due to coacervation (𝚫*Q*_c_). Figure 2a shows a sample ITC curve for PCM formation. DLS correlation functions show monodisperse PCMs and SAXS curves fit to a spheroidal form factor show good agreement with experimental data at the salient length scales.

In the IP model for polyelectrolyte complexation, binding between oppositely charged monomers occurs independently of other sites. As a result, rather than using polymer concentrations, we use monomer concentrations of polycation and polyanion here. All ITC curves are therefore shown in terms of energy per lysine monomer as a function of the molar ratio between the cationic species (Lys) and anionic species (Glu). Thermodynamic quantities extracted from the model fits are also analyzed on a per monomer basis. Presenting data in this fashion also allows for a direct comparison between BCPs containing different cationic and neutral block lengths.

Structural features regarding any systems that formed monodisperse PCMs were measured using DLS, MALS, SAXS, and transmission electron microscopy (TEM). In particular, DLS was used to determine the PCM hydrodynamic radius (*R*_h_), SAXS was used to determine PCM BCP aggregation number (*n*_BCP_) and core radius (*R*_core_), MALS was used to confirm *n*_BCP_, and TEM was used to visualize PCM cores for their morphology. Sample DLS and SAXS curves are shown in Figure 2b,c, respectively. DLS correlation functions showed monodisperse PCMs and SAXS curves fitted to a spheroidal form factor showed good agreement with experimental data at the salient length scales. A full list of fitting parameters for DLS and SAXS experiments can be found in Table S5. Trends in structural data were then examined for possible underlying explanations for the thermodynamic data collected.

### Complexation Free Energy Contributions for Matched Charged Block Lengths

**Figure 3.**
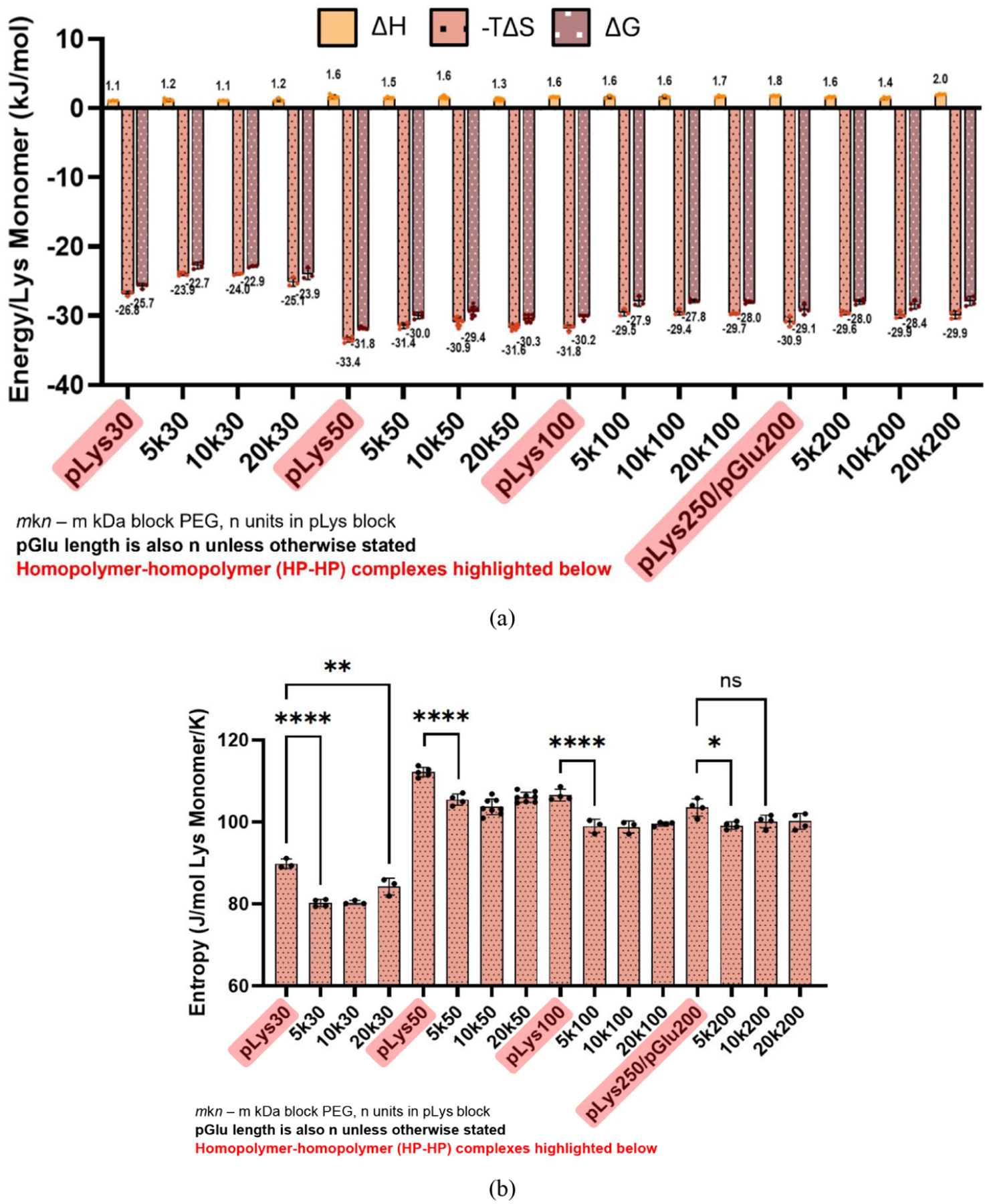
(a) Free energy contributions to complexation on a per monomer basis after fitting ITC data to the IP model for matched charged block length complexes. Values from individual trials are shown with dots. As expected, complexation occurs even though the process is endothermic because of the largely entropic driving force. Homopolymer–homopolymer complexes are highlighted at the bottom in red. (b) Entropy values for complexation of matched charged block length complexes with statistical comparisons between block copolymer–homopolymer complexation (BCP–HP) and homopolymer–homopolymer (HP–HP) complexation (ANOVA test for each charged block length group). The results show that BCP–HP complexation is significantly less entropically stabilized than HP–HP complexation, especially at low concentration. A complete list of statistical comparisons can be found in Figure S2.

To narrow the parameter space of block length combinations examined, we first focused on systems with matched charged block lengths for the homopolymer (HP) pGlu and the pLys block. The length of the charged block (Lys and Glu) was varied between 30 units and 200 units, while the PEG block molecular weight (MW) was varied between 5 kDa and 20 kDa. Figure 3a shows the complexation free energy contributions for each combination of BCP and HP tested. For comparison, the energies corresponding to homopolycation–homopolyanion complexation for the same charged block lengths were also measured (highlighted in red at the bottom in Figure 3), with the exception of pLys250/pGlu200 complexes, where obtaining pLys200 was not possible. In all cases, we see that complexation is slightly endothermic or enthalpically unfavorable. However, we see that this enthalpic barrier is overcome by the largely entropically driven complexation as substantiated by previous experimental and theoretical work.^1,20,21,27^ The differences in free energy among the various complexes are also largely due to entropic differences.

### Complexation Entropy for Matched Charged Block Lengths

For ease of comparison, Figure 3b shows just the entropy of complexation (having divided by −*T*_ITC_ = −298 K) along with the statistical significance of relevant comparisons. Further details regarding statistical comparisons between the entropy results can be found in Figure S2. When comparing the entropy gained in BCP–HP complexation and HP–HP complexation for a given charged block length, we see significant differences due to the presence of the PEG block attached to the pLys. However, changes in entropy are less pronounced among complexes formed with different PEG block MWs for a constant charged block length. To see this comparison more readily, we monitor the entropic penalty (𝚫*S*_penalty_) of BCP–HP complexation with respect to the baseline of HP–HP complexation for a given charged block length, as given by eq 4. 𝚫*S*_penalty_ from BCP–HP complexation can be understood as the consequence of having to tether the PEG block to the core of a complex, since the PEG block does not participate in the complexation mechanism.


(4) $$𝚫S_{\text{penalty}}=𝚫S_{\text{HP}-\text{HP}}-𝚫S_{\text{BCP}-\text{HP}}$$

Table 1 shows how the entropic penalty varies with PEG block MW and charged block length. For all charged block lengths, 𝚫*S*_penalty_ is independent of PEG block MW. However, the presence of a PEG block is enough to induce a uniform entropic penalty for BCP–HP complexation. Additionally, we see a general decrease in as a function of charged block length. While the presence of a PEG block attached to the cationic block incurs an entropic penalty, the magnitude of this penalty depends on the charged block length. In general, the bigger the charged blocks involved in the complexation, the smaller the entropic penalty of dragging the PEG chain to form a complex.

Interestingly, when combining polymers with charged block lengths of 30 together in solution, DLS does not show the formation of monodisperse PCMs, yet the observed trends for 𝚫*S*_penalty_ largely hold. The inability of polymers with charged block lengths of 30 to form monodisperse PCMs may be explained by their relatively low enthalpies and entropies of complexation (Figure 3a). Below a certain threshold, the charged block length may not be long enough to produce the electrostatic interactions and entropic driving force necessary for the microscopic phase separation that results in PCM formation. However, there is still an entropic penalty for having to drag a PEG block since ion pairing between individual polymers occurs in these systems, which justifies why the trend in 𝚫*S*_penalty_ holds even below the charged block length threshold for PCM formation.

Another notable exception in the data is that 𝚫*S*_penalty_ seems to increase with charged block length when the neutral block is much larger than the charged blocks, as seen in the 20 kDa PEG block data set for charged block lengths below 100. This difference may be explained by previous studies, which predict a gradual transition from star-like to crew-cut PCMs as PCM corona thickness decreases in relation to the core size.^28–30^ Though the strict definitions of these regimes vary, these studies show that as the corona-forming neutral block size increases in relation to the core-forming charged blocks, the PCM aggregation number decreases due to the crowding of longer neutral chains around a smaller charged core. Furthermore, PCMs may exhibit a less dense core due to a decrease in ion pairing. Both trends are substantiated by scattering data and scaling laws (Tables 4–6) discussed in later sections. Below a certain block length, the decreased aggregation number, core density, and extent of ion pairing may result in substantially looser PCM aggregates, allowing for more movement, and perhaps increased chain exchange rates in these systems. In other words, the entropic penalty incurred from the tethered PEG block in BCP–HP complexation as compared with HP–HP complexation is partially offset by the potential gain in translational entropy of the constituent chains in systems with large corona-forming blocks and small core-forming blocks.

For those combinations of BCPs and HPs that did form PCMs, we compare their entropy of complexation, the results of which are in Table 2. Notably, we see a decrease in the entropy of PCM formation as the charged block length increases, particularly from a block length of 50 to 100. We will comment on this result later when discussing PCM structural features such as PEG chain density and brush height. Both Tables 1 and 2 show that while complexation is sensitive to charged block length, it remains largely unaffected by PEG block length. This would indicate some effect originating from the charged core of the PCM.

### Complexation Entropy for Mismatched Charged Block Lengths

In this section, we vary the charged block length of the BCP and HP independently and study the effect on complexation entropy. Monodisperse PCMs formed for all combinations of charged block lengths between 50 and 200 as indicated by DLS data shown in Table S5 by combining neutral-cationic PEG-pLys BCPs and anionic pGlu HPs at equal monomer concentrations (charge ratio of unity). Data for 𝚫*H*, −*T*𝚫*S*, and 𝚫*G* are included in Table S4. As is the case with matched charged block lengths, BCP–HP complexation with mismatched charged block lengths is slightly enthalpically unfavorable and highly entropically driven. As a result, we perform a similar analysis as before, focusing on the entropy of complexation, yielding the results in Table 3, with the original data for matched charged block length complexes (Table 2) shown along the diagonal.

From these data, we see that once again, the entropic driving force for complexation is independent of PEG block MW for all charged block length combinations. We also see a dependence of the entropy on the charged block length, and are further able to parse the effects of the polycationic block (pLys) and the homopolyanion (pGlu). PCMs formed from the smallest pGlu HP length (pGlu50) and the largest pLys block length (pLys200) on the BCP exhibit the highest entropy of complexation. On the other hand, the lowest entropies are measured for large pGlu HP length (pGlu200) and small pLys block length (pLys50). The entropy of complexation is also more sensitive to changes in pGlu HP length than pLys block length. The stronger dependence on pGlu HP length is shown in the matched block length entropy data (Table 2).

**Figure 4.**
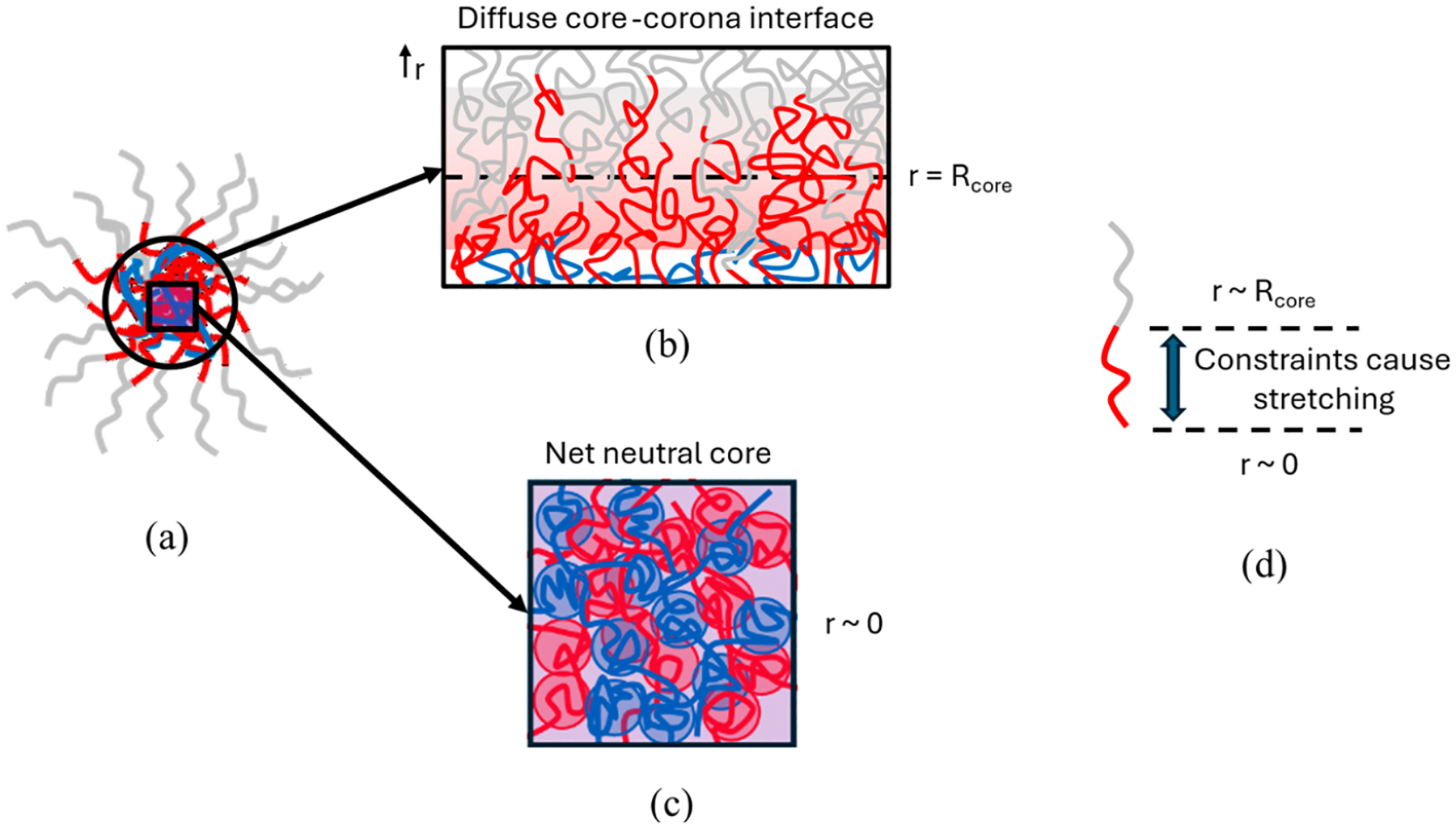
Schematic illustrating the hypothesized structure of PCMs and the interactions that impose constraints on the PEG-pLys BCP. (a) Overall PCM structure composed of a dense, charged core composed of pLys (red) blocks and pGlu (blue) polyanions surrounded by a corona composed of PEG (gray). (b) A magnified view of the hypothesized behavior of BCP chains near the core–corona interface illustrating the proposed diffuse nature of the core–corona interface around *r* = *R*_core_. When PCM self-assembly occurs, one constraint is that the BCP junction between the pLys and PEG blocks is forced to be near the core–corona interface separating charged and uncharged monomers. However, the distribution of radial positions of junctions is broad and the transition from charged to uncharged domains is gradual. We discuss the nature of this interface in further detail in the ensuing sections. (c) A magnified view of the behavior of polymer chains within the charged, net-neutral core of the PCM. Here, the second constraint on the BCP is that electrostatic repulsion suppresses long-range polyelectrolyte fluctuations, entailing that the pLys block forms ion pairs to neutralize the pGlu chain. (d) The constraints illustrated in (b) and (c) force smaller pLys blocks into entropically less favorable, stretched conformations. A larger pLys block can span the entire core radius without taking on such a conformation, readily satisfying both constraints.

One possible reason for this asymmetry in charged block length dependency is that while pGlu is a HP, the pLys block is a component of the BCP, meaning that it is attached to the PEG block that composes the corona of the PCM. Figure 4 as a whole shows a sequence of schematics that illustrate the structural basis for the asymmetry, with Figure 4a showing a macroscopic illustration of PCM structure. Unlike in the case of pGlu, being attached to the corona block implies that there would be a slight outward bias for pLys to be present toward the outer part of the charged core (Figure 4b).^31^ However, for a stable PCM to form, there would also have to be enough ion pairs throughout the core to suppress long-range polyelectrolyte fluctuations, resulting in an inward bias (Figure 4c). A longer charged block on the BCP would more readily be able to satisfy both requirements without having to take on an entropically unfavorable, stretched conformation (Figure 4d). Likewise, a shorter charged HP would also allow for this balance to occur more readily, since it would be encapsulated in the core.

### PCM Core Radius Size (*R*_core_)

We conducted SAXS experiments to measure charged PCM core size to explain the dependency of the entropic driving force on the sizes of the charged blocks. Given that the scattering length density (electron density) of the PEG corona is close to that of the PBS/water solvent, the corona is effectively transparent in this measurement after background subtraction. Calculations pertaining to the scattering length density contrasts of each of the components can be found in Table S8.

Table 4 shows the results of the SAXS data fit to a spheroidal form factor with aspect ratios ranging from 1 to 2, indicating nearly spherical particles, substantiated by TEM images (sample image shown in Figure 2d). The *R*_core_ data are then fit to power laws (Table 5) and compared against PCM scaling laws fit to results from a previous study for a similar system.^24^ While preparation methods slightly differ and the salt concentration in our study is much larger (160 mM ionic strength vs 55 mM ionic strength), we see that the relative strength of the dependencies on polymer block lengths is consistent. We see a stronger dependence of *R*_core_ on the length of the pLys block (*N*_Lys_^0.57±0.03^), followed by a weaker dependence on the length of the PEG block (*N*_PEG_^−0.25±0.02^), and no dependence on the length of the pGlu polyanion (*N*_Glu_^−0.01±0.03^). Treating water as a good solvent with (Flory exponent *ν* = 0.588), the scalings from both studies are also roughly in line with previously developed scaling theories for PCM structure.^28,30^ The weaker dependence of PCM size on pLys block length across all attributes compared to the previous study can be attributed to the increased salt concentration at which these micelles were prepared. The increased ionic strength could screen out charge–charge interactions and their effects on PCM structure to a greater degree.

### Aggregation Number for BCPs in a PCM (*n*_BCP_)

Aggregation numbers (*n*_BCP_) for PCMs are calculated from SAXS data as done previously,^24^ from the forward scattering intensity (*I*(*q* = 0)) using eq 5, where 𝚫*ρ*_*core*_ is the scattering length density contrast of the core (details of SLD calculation in Table S8) with respect to the solvent (PBS/H_2_O), and *c*_BCP_ is the number concentration of BCPs in solution. This formula importantly assumes that the free polymer in solution is negligible compared to the concentration that is complexed. MALS was used to check for the validity of this assumption by independently measuring the molecular weight of formulated PCMs through Zimm analysis, without the need for this assumption. From the molecular weight, *n*_BCP_ for MALS is calculated (Table S6) assuming overall charge neutrality of the PCMs, which has been shown in previous studies.^32^ The calculated *n*_BCP_ from both SAXS and MALS data show good agreement (Figure S3), supporting the assumption that the majority of polymers are found in PCMs. CBQCA, a fluorescence-based, peptide quantification assay, was also run on select filtered micelle samples to ensure that the majority of polymers (polypeptides in this system) were within a complex (Table S7), further validating the ensuing analyses of the SAXS data through eq 5

(5) $$n_{\text{BCP}}=\frac{V_{\text{core}}^{2}𝚫\rho_{\text{core}}^{2}c_{\text{BCP}}}{I(q=0)}$$

The forward scattering intensity is extrapolated from a linear fit of a Guinier plot (ln(*I*) vs *q*^2^) in the region where 0.5 < *qR*_*g*_ < 1.3. Table 6 shows the results of this analysis and Table 5 shows the scaling dependencies of *n*_BCP_ on the block lengths of the constituent polymers. Here, we see a negative dependence of *n*_BCP_ on PEG block length (*N*_PEG_^−1.03±0.17^), and a weaker, positive dependence on pLys block length (*N*_Lys_^0.85±0.19^). Both dependencies amount to aggregation numbers between 10^1^–10^3^ per PCM for the polymers tested.

### PEG Chain Surface Density (*σ*) and Corona Brush Height (*h*)

Initially, we wanted to consider the conformation of the corona chains as the cause for the differences in the entropies of complexation between PCM formulations. Previous studies using self-consistent field theory (SCFT)^33^ have shown that chains tethered to a curved interface, as would approximately be the case with the PEG corona chains being tethered to a charged core, take on an extended conformation at low degrees of interfacial curvature (or at high radii of curvature). For a given chain surface density, the polymer brush height increases as the extra degree of freedom for the tethered polymers to splay is reduced from a curved to flat interface. Increasing the surface chain density on the charge core yields a similar effect due to crowding. Figure S4 shows a schematic of this phenomenon as it would apply to PCMs. Briefly, if the PEG chain conformation within the corona were to affect the entropy of complexation, then all else being equal, the entropy of a highly stretched PEG chain on a flatter core–corona interface (larger *R*_core_) would be less than that of a splaying PEG chain on a highly curved core–corona interface (smaller *R*_core_). Table 2 for matched charged block length PCMs suggests this to be the case since there is a decrease in the 𝚫*S* of PCM formation as the charged block length, a proxy for *R*_core_, increases.

We next examine if this trend between *R*_core_ and 𝚫*S* for matched charged block length PCMs is correlated with the PEG chain surface density (*σ*) or brush height (*h*). PEG chain surface density is calculated by dividing the BCP aggregation numbers *n*_BCP_ by the charged core surface area ($4\pi R_{\text{core}}^{2}$). The corona brush height is calculated by taking the difference between *R*_h_ as measured by DLS and *R*_core_ as measured by SAXS.

**Figure 5.**
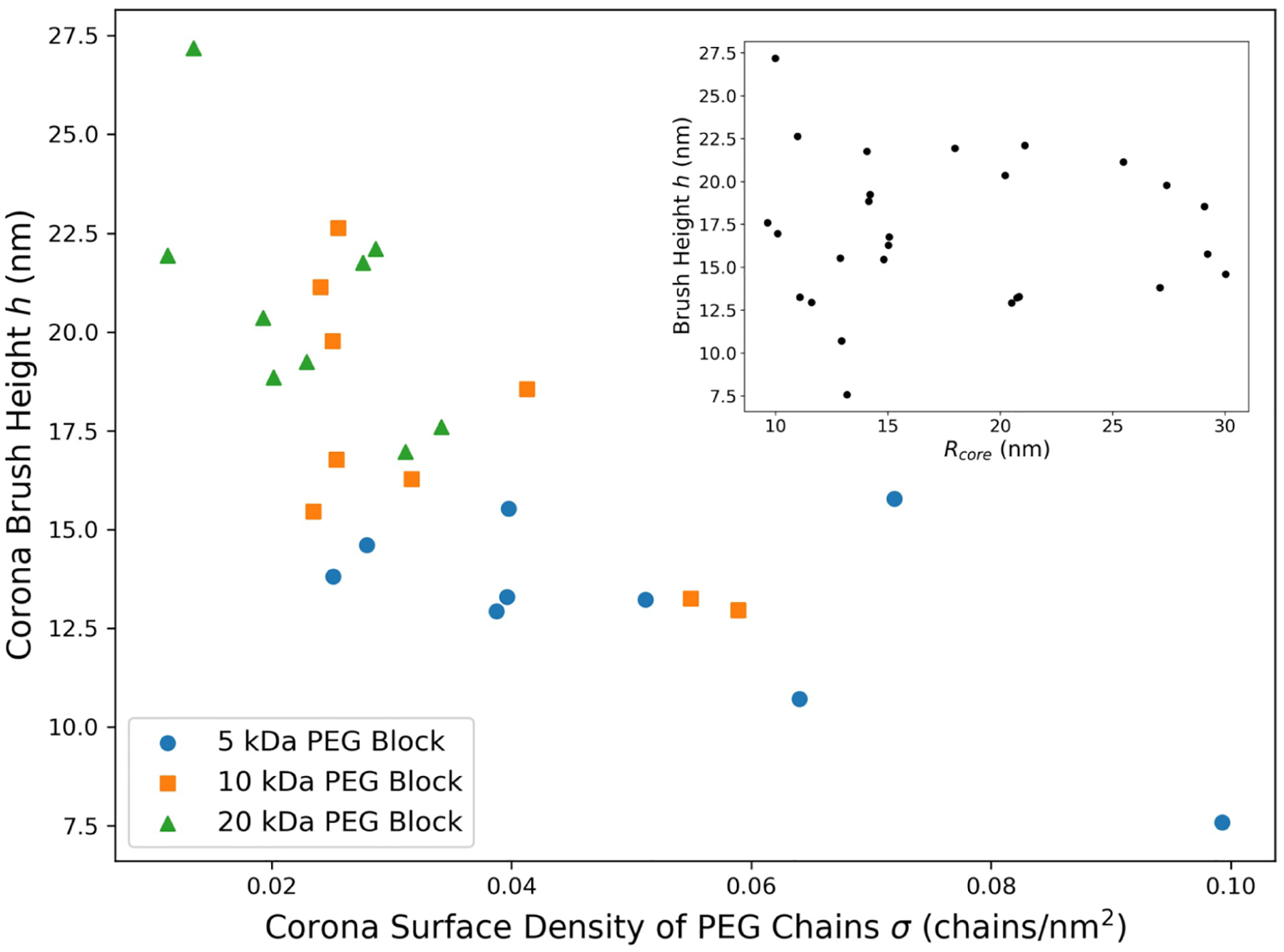
Corona brush height (*h*) as a function of corona surface density of PEG chains (*σ*) for all PCM combinations (mismatched and matched charged block length). For all PEG block lengths, we see a negative correlation between *h* and *σ* which is different from the positive correlation that would be predicted based on a system of chains tethered to a curved interface (Figure S4). It seems that as chain density increases, corona PEG chains take on a less extended conformation. These results are not correlated with the core radius as shown in the inset.

Interestingly, there is no correlation between *σ* and 𝚫*S* and, unexpectedly, a slight positive correlation between *h* and 𝚫*S* (Figure S5 and Figure S6). The correlation with *h* is slightly more apparent in matched charged block length PCMs (Figure S6a) than in mismatched charged block length PCMs (Figure S5a) when separated based on PEG block MW. For the same PEG size, an extended chain length implies a higher entropy of complexation, which would suggest that PEG chain conformation/extension is not determining the overall thermodynamics of PCM formation based on the previously mentioned hypothesis. In fact, we also find that *σ* and *h* are negatively correlated (Figure 5) with each other across all charged block length combinations (taking into account various PEG MWs), which suggests that as PEG chains become more crowded along the core–corona interface, the brush height decreases. Furthermore, *R*_core_ does not correlate with *h* as shown in the inset of Figure 5. These correlations further suggest that both PCM thermodynamics and PCM structural trends are not governed by the reasoning outlined previously, most likely because of the self-assembled nature of the PCM. First, both the structural and thermodynamic trends are also governed by the complexation that occurs in the PCM core, which in the case of the SCFT model is an inert, smooth surface. In addition, the SCFT model assumes that 1) *σ* is a controllable parameter in PCMs that exceeds the density threshold necessary for neighboring chains to squeeze each other, and 2) the surface (core–corona interface) is a rigid boundary from which all PEG chains originate. Neither is likely the case in PCMs.

Regarding the first assumption, previous studies have quantified polymer chain crowding using reduced tethering density ($\tilde{\sigma }=\sigma \pi {R_{g}}^{2}$), which is best understood as the number of tethered chains within the same area as a chain in an unperturbed conformation in the same solvent (π*R*_*g*_^2)^.^34–36^ Here, *R*_*g*_ is the radius of gyration of unperturbed PEG in a good solvent, and can be calculated based on PEG block molecular weight.^34^ The calculated values are shown in Table S9.

Studies have shown that up until $\tilde{\sigma }\sim 3-4$, tethered brushes are not crowded enough to be squeezed by their nearest neighbors and display the behavior depicted in Figure S4.^34,35^ As shown in Table S9, the majority of PCM formulations do not exhibit the necessary density of PEG chains to meet this threshold, which would explain why the entropy of complexation is not determined by PEG chain conformation. However, below this threshold, the expectation would be that for a given PEG block length, *h* would be relatively constant with respect to *σ*. Here, the negative correlation between *σ* and *h*, even within each PEG molecular weight data set, would suggest another reason for the discrepancy, namely the second assumption made in the SCFT model regarding the core–corona interface.

Here, the seemingly contradictory nature of the structural data strongly suggests that the boundary between the core and corona of a PCM is diffuse as drawn in Figure 4b. The calculation of chain surface density *σ* and corona brush height *h* would change in this picture. PEG chains would originate from a range of radial positions *r = R*_core_ ± *δ*, which would change the height of each chain in the brush. Furthermore, an increasingly diffuse interface would render the area calculation used to determine surface chain density inaccurate and limit the applicability of crowding arguments due to the increased freedom afforded to BCP junctions. To assess the validity of Figure 4b, further scattering experiments would be needed to probe for the interfacial surface fractal for each formulation.^37,38^ Alternatively, the incorporation of a more detailed form factor to account for an interface with a certain width or gradient might add more insight.^39^ Both approaches would yield information that would alter the analysis in Figure 5, perhaps accounting for the negative correlation between *σ* and *h*.

The reasoning for hypothesizing the existence of a diffuse core–corona interface is that compared to most micelle systems, where interfacial tension is governed by the presence of two immiscible phases and self-assembly is governed by the hydrophobicity of two domains, the interfacial tension in PCM systems between the charged core and neutral corona is small since the entire micelle system is hydrophilic and highly hydrated.^40^ The minimal difference in solvent affinity between the core-forming and corona-forming blocks of a PCM suggests that the interfacial boundary in a PCM should not be treated in the same manner as in typical micelle systems, where the core–corona interface is well-defined. In reality, the PCM core can be likened to a liquid coacervate droplet that is colloidally stabilized by the PEG block. The PEG block sterically hinders the phase separation process, but does not change the nature of the diffuse, permeable interface of the droplet, which delineates the boundary between charged and uncharged domains.

### Core Ion Pair Density (*ϕ*_IP_) and Corona PEG Monomer Density (*ϕ*_PEG_) in Matched Charged Block PCMs

**Figure 6.**
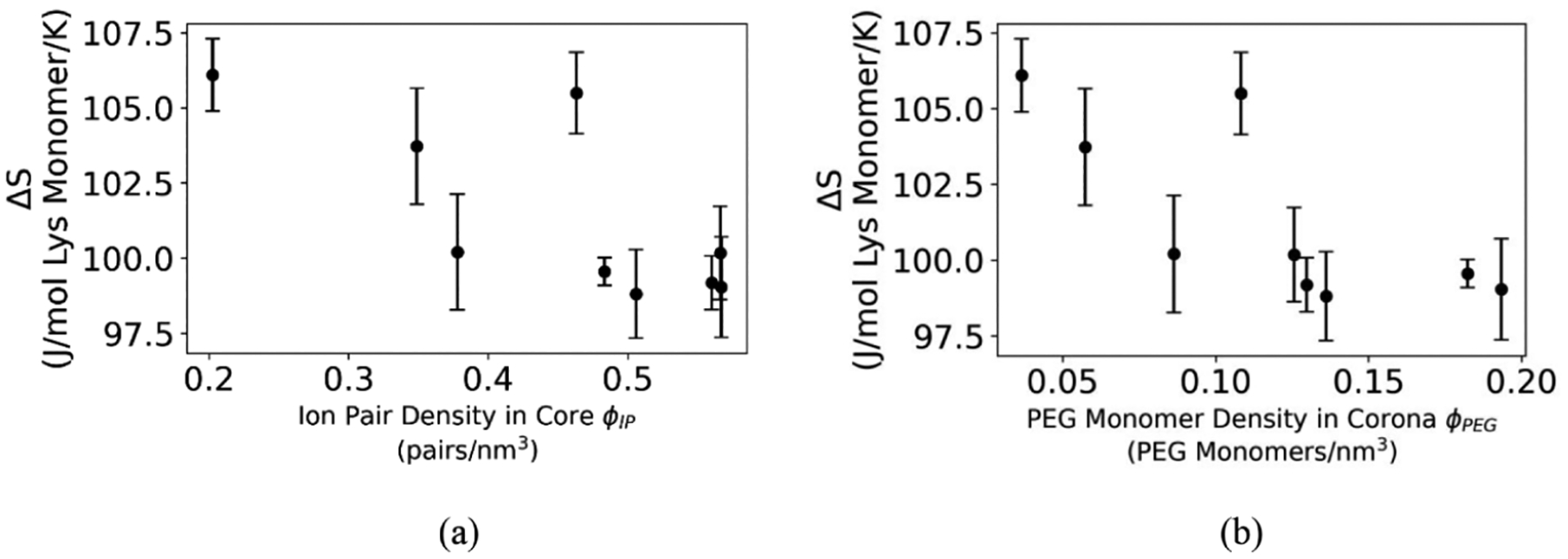
(a) Entropy of complexation, 𝚫*S*, as a function of ion pair density, *ϕ*_IP_, within the core of a matched charged block length PCM. *ϕ*_IP_ is calculated as per eq 6, using SAXS forward scattering data (*I*(*q* = 0)) obtained from a Guinier fit. *ϕ*_IP_ is negatively correlated with entropy of complexation in PCMs, perhaps indicating crowding effects that limit the translation entropy of individual polymer chains. (b) Entropy of complexation, 𝚫*S*, as a function of PEG monomer density, *ϕ*_PEG_, in the corona of a matched charged block length PCM. *ϕ*_PEG_ is calculated as per eq 7, using the same SAXS forward scattering data. *ϕ*_PEG_ is also negatively correlated with 𝚫*S*, suggesting similar steric effects could limit chain translational entropy, causing an overall loss in entropy of complexation.

To examine the core, we calculate the ion pair density (*ϕ*_IP_), which is given by eq 6, where *N*_PEG_ is the unit length of the PEG block. For matched charged block length PCMs, we see that *ϕ*_IP_ is negatively correlated with 𝚫*S* (Figure 6a), which indicates that as the core becomes more dense, complexation becomes entropically less favorable, akin to an argument around crowding within the core. As the core becomes denser, the translational entropy of the polymer chains making up the PCM should decrease. Calculations for the PEG monomer density (*ϕ*_PEG_) in the corona, given by eq 7, show the same negative correlation with 𝚫*S* (Figure 6b). Both trends suggest that denser cores and coronas lead to lower entropies of complexation in matched charged block length systems.


(6) $$\phi_{\text{IP}}=\frac{n_{\text{BCP}}N_{\text{Lys}}}{\frac{4\pi }{3}R_{\text{core}}^{3}}$$

(7) $$\phi_{\text{PEG}}=\frac{n_{\text{BCP}}N_{\text{PEG}}}{\frac{4\pi }{3}\left(R_{h}^{3}-R_{\text{core}}^{3}\right)}$$

## CONCLUSIONS

Understanding the thermodynamic driving forces involved in polyelectrolyte complexation is crucial for the rational design of PCMs with optimal self-assembly properties, particularly in the context of drug delivery. Here, we perform a comprehensive thermodynamic and structural analysis of PCMs, examining the effect of varying component polymer block lengths on the entropic driving force for complexation, and using structural data to inform these trends. The experimental results in this paper show that complexation in PCMs is largely entropically driven by the charged blocks that make up the core of the micelle. Meanwhile, the addition of a corona PEG block in PCMs, when compared with homopolyelectrolyte analogues, incurs a uniform entropic penalty with respect to PEG block length that decreases with the charged core block lengths. Structural data suggest that core ion pair density and corona monomer density, not PEG chain conformation, play a role in the entropy of complexation. These correlations suggest that the entropy of PCM formation may be affected by the translational entropy of the polymer chains constituting the PCM, and that the PCM core–corona interface may be more diffuse than that of a typical micelle surfactant system. Future experiments will be required to analyze PCMs formed from different polymer components, whether for two diblock copolymer micelles (AB + AC or AB + CD where A, D are neutral and B, C are charged) PCMs, or for BCP–HP PCMs formed with different homopolyanions of therapeutic relevance. Such experiments would prove the extent to which these findings are applicable for PCM complexation in general across a variety of polymer platforms and configurations.

Given the interest in using neutral-cation BCPs as drug delivery agents for oligonucleotide cargos, current efforts in our lab are utilizing the same analytical framework to examine BCP complexation with RNA/DNA oligonucleotides varying in structure, sequence, and size. As mentioned before, pGlu was the polyanion cargo of choice for this study due to its simpler polymer physics. For oligonucleotide cargos, a myriad of factors can alter the polymer physics underpinning PCM selfassembly. Oligonucleotides are composed of aromatic bases that can engage in cation-pi interactions with polycations, as well as intermolecular pi–pi stacking interactions with other bases.^41^ Oligonucleotide rigidity can be affected by whether it is single-stranded or double-stranded.^42^ The diversity in oligonucleotide sequences can also lead to a host of secondary structures (loops, junctions, etc.).^43,44^ Secondary structure can also significantly alter phosphate backbone charge density,^42^ which is a crucial parameter when defining polyelectrolyte systems. Previous studies have partially examined this parameter space,^25,30,45^ but a better understanding of polyelectrolyte interactions within PCMs is needed to inform BCP design for the delivery of an increasing library of oligonucleotides with therapeutic potential.

## Supplementary Material

Supplementary Info

The Supporting Information is available free of charge at https://pubs.acs.org/doi/10.1021/acs.macromol.5c03639.

Additional data and experimental parameters from ITC, SAXS, DLS, CBQCA, and MALS; additional plots concerning thermodynamic data and the relationship with various PCM features; polymer characterization (PDF)

## Figures and Tables

**Table 1.** Entropic Penalty for Matched Charged Block Length Complexes^a^

Δ*S*_penaity_ (J mol Lys^−1^ K^−1^)	Charged Block Length (both pLys and pGlu)			
PEG Block MW (kDa)	30	50	100	200
5	9.6 ± 1.4	6.7 ± 1.8	7.6 ± 2.2	4.4 ± 2.3
10	9.5 ± 1.2	8.5 ± 2.2	7.8 ± 2.0	3.4 ± 2.7
20	5.6 ± 2.4	6.1 ± 1.7	7.0 ± 1.5	3.4 ± 2.9

a The entropic penalty remains relatively uniform across different PEG block sizes and generally decreases as charged block length increases.

**Table 2.** Entropy of Complexation for Matched Charged Block Length BCP–HP Complexes That Led to Monodisperse PCM Formation^a^

Δ*S* (J mol Lys^−1^ K^−1^)	Charged Block Length (both pLys and pGlu)		
PEG Block MW (kDa)	50	100	200
5	105.5 ± 1.4	99.0 ± 1.7	99.2 ± 0.9
10	103.7 ± 1.9	98.8 ± 1.5	100.2 ± 1.6
20	106.1 ± 1.2	99.6 ± 0.5	100.2 ± 1.9

a There is a general decrease in entropy as charged block length increases.

**Table 3.** Entropy of Complexation 𝚫*S* (J mol Lys^−1^ K^−1^) for All Combinations of Block Lengths That Led to Monodisperse PCM Formation

pLys Block Length	PEG Block MW (kDa)	pGlu50	pGlu100	pGlu200
50	5	105.5	100.3	92.8
	10	103.7	101.4	93.5
	20	106.1	98.5	93.0
100	5	105.9	99.0	98.6
	10	107.2	98.8	98.4
	20	104.4	99.6	97.8
200	5	108.9	102.5	99.2
	10	109.3	102.7	100.2
	20	107.7	103.2	100.2

**Table 4.** PCM Core Radius *R*_core_ (nm) for All Combinations of Block Lengths That Led to Monodisperse PCM Formation

pLys Block Length	PEG Block MW (kDa)	pGlu50	pGlu100	pGlu200
50	5	12.9	12.9	13.2
	10	11.0	11.6	11.1
	20	10.0	10.1	9.6
100	5	20.8	20.5	20.7
	10	14.8	15.1	15.0
	20	14.1	14.2	14.1
200	5	29.2	30.0	27.1
	10	27.4	29.1	25.5
	20	21.1	20.2	18.0

**Table 5.** Scaling Power Law Dependencies for PCM Structural Attributes as a Function of Block Lengths of Constituent Polymers (*N*_PEG_, *N*_Lys_, *N*_Glu_)^a^

Variable	*R*_core_	*n*_BCP_	*R*_h_	*h*
*N*_PEG_	−0.25 ± 0.02	−1.03 ± 0.17	0.04 ± 0.04	0.34 ± 0.06
*N*_Lys_	0.57 ± 0.03	0.85 ± 0.19	0.35 ± 0.04	0.15 ± 0.07
*N*_Glu_	−0.02 ± 0.03	0.07 ± 0.18	−0.08 ± 0.04	−0.13 ± 0.07
*N*_PEG_	−0.17 ± 0.04	−0.74 ± 0.31	0.17 ± 0.06	0.53 ± 0.21
*N*_Lys_	0.73 ± 0.11	1.37 ± 0.41	0.51 ± 0.16	0.25 ± 0.26
*N*_Glu_	−0.05 ± 0.06	−0.21 ± 0.25	−0.02 ± 0.07	–

a The first set of scalings pertain to our results for PCMs prepared in 1× PBS (ionic strength ≈ 160 mM). The second set of scalings pertain to results from Marras et al.^24^ where PCMs were prepared via dialysis in 10 mM HEPES and 50 mM NaCl (ionic strength ≈ 55 mM).

**Table 6.** BCP Aggregation Number *n*_BCP_ as Calculated from SAXS Data for All Combinations of Block Lengths That Led to Monodisperse PCM Formation

pLys Block Length	PEG Block MW (kDa)	pGlu50	pGlu100	pGlu200
50	5	83	135	217
	10	39	100	85
	20	17	40	40
100	5	216	205	277
	10	65	72	90
	20	51	58	69
200	5	772	316	233
	10	237	439	196
	20	160	99	46
