# Supplementary material for: Effect of Polymer Structure on the Thermodynamics of Polyelectrolyte Complex Micelle Formation
Source: Macromolecules. Author manuscript; Available in PMC 2026 Apr 18. (PMC13089909; doi:10.1021/acs.macromol.5c03639)
Supplement: Supplementary Info [file NIHMS2161913-supplement-Supplementary_Info.pdf]

# Supplementary Information:

# Effect of Polymer Structure on the Thermodynamics of Polyelectrolyte Complex Micelle Formation

Vishnu L. Dharmaraj,[†] Yun Fang,[‡] and Matthew V. Tirrell[*,†]

*†Pritzker School of Molecular Engineering, ‡Biological Sciences Division, Department of Medicine, University of Chicago, Chicago, IL 60637*

E-mail: mtirrell@uchicago.edu

For the entirety of the supplementary information, we refer to a block copolymer (BCP) containing a PEG block with molecular weight (MW) of *m* kDa and a pLys block of *n* Lys units as a *m*k*n* BCP.

**Polymer Characterization**

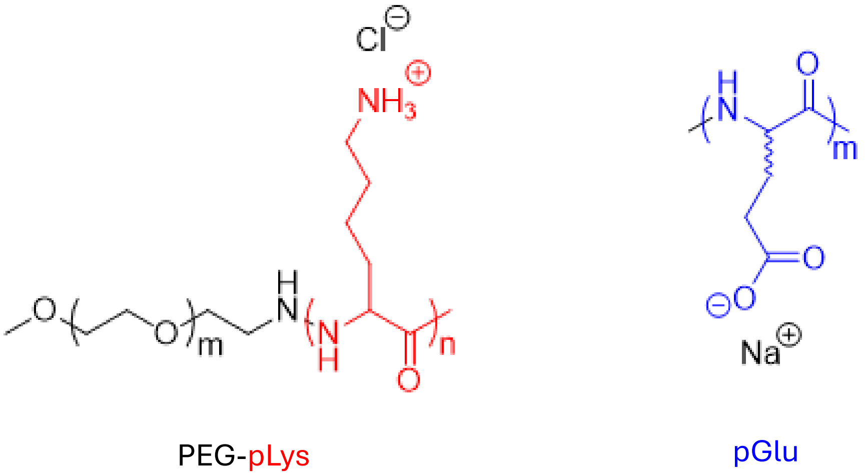


Figure S1: Chemical structure for methoxy-poly(ethylene glycol)-block-poly(L-lysine hydrochloride) (PEG-pLys), poly(D,L-glutamic acid) sodium salt (pGlu)

Table S1: Molecular weight specifications for polymers tested.

| Sample | PDI by GPC | MN mPEG-NH2 (kg/mol) | MN by NMR (kg/mol) | Charged DP |
|---|---|---|---|---|
| pLys30 | 1.02 | – | 5.4 | 33 |
| pLys50 | 1.05 | – | 9.0 | 55 |
| pLys100 | 1.03 | – | 16.5 | 100 |
| pLys250 | 1.07 | – | 41.0 | 234 |
| pGlu30 | 1.03 | – | 5.0 | 33 |
| pGlu50 | 1.01 | – | 7.9 | 52 |
| pGlu100 | 1.03 | – | 16.6 | 110 |

| Sample | PDI by GPC | MN mPEG-NH2 (kDa) | MN by NMR (kDa) | Charged DP |
|---|---|---|---|---|
| pGlu200 | 1.02 | – | 29.6 | 196 |
| 5k30 | 1.03 | 5.0 | 9.4 | 27 |
| 10k30 | 1.03 | 10.3 | 14.7 | 27 |
| 20k30 | 1.09 | 21.0 | 25.6 | 28 |
| 5k50 | 1.06 | 5.3 | 13.0 | 47 |
| 10k50 | 1.04 | 10.3 | 17.9 | 46 |
| 20k50 | 1.07 | 20.0 | 27.6 | 46 |
| 5k100 | 1.05 | 4.6 | 21.2 | 101 |
| 10k100 | 1.06 | 10.3 | 25.6 | 93 |
| 20k100 | 1.02 | 21.0 | 37.3 | 99 |
| 5k200 | 1.06 | 5.0 | 40.4 | 194 |
| 10k200 | 1.03 | 10.3 | 40.6 | 184 |
| 20k200 | 1.03 | 20.0 | 54.9 | 212 |

**Sample Concentrations**

**ITC Concentrations**

Table S2: Charged monomer concentrations utilized for ITC testing.

| Polycation | Polyanion | Cationic Monomer Concentration (mM) | Anionic Monomer Concentration (mM) |
|---|---|---|---|
| pLys30 | pGlu30 | 9.90 | 0.62 |
| 5k30 | pGlu30 | 8.10 | 0.62 |
| 10k30 | pGlu30 | 8.10 | 0.62 |
| 20k30 | pGlu30 | 8.40 | 0.62 |
| pLys50 | pGlu50 | 4.40 | 0.26 |
| 5k50 | pGlu50 | 3.76 | 0.26 |
| 10k50 | pGlu50 | 3.68 | 0.26 |
| 20k50 | pGlu50 | 3.68 | 0.26 |
| pLys100 | pGlu100 | 8.00 | 0.55 |
| 5k100 | pGlu100 | 8.08 | 0.55 |
| 10k100 | pGlu100 | 7.44 | 0.55 |
| 20k100 | pGlu100 | 7.92 | 0.55 |
| pLys250 | pGlu200 | 7.49 | 0.49 |
| 5k200 | pGlu200 | 7.76 | 0.49 |
| 10k200 | pGlu200 | 7.36 | 0.49 |
| 20k200 | pGlu200 | 8.48 | 0.49 |
| 5k50 | pGlu100 | 3.76 | 0.28 |
| 10k50 | pGlu100 | 3.68 | 0.28 |
| 20k50 | pGlu100 | 3.68 | 0.28 |
| 5k50 | pGlu200 | 7.52 | 0.49 |

| Polycation | Polyanion | Cationic Monomer Concentration (mM) | Anionic Monomer Concentration (mM) |
|---|---|---|---|
| 10k50 | pGlu200 | 7.36 | 0.49 |
| 20k50 | pGlu200 | 7.36 | 0.49 |
| 5k100 | pGlu50 | 8.08 | 0.52 |
| 10k100 | pGlu50 | 7.44 | 0.52 |
| 20k100 | pGlu50 | 7.92 | 0.52 |
| 5k100 | pGlu200 | 8.08 | 0.49 |
| 10k100 | pGlu200 | 7.44 | 0.49 |
| 20k100 | pGlu200 | 7.92 | 0.49 |
| 5k200 | pGlu50 | 7.76 | 0.52 |
| 10k200 | pGlu50 | 7.36 | 0.52 |
| 20k200 | pGlu50 | 8.48 | 0.52 |
| 5k200 | pGlu100 | 7.76 | 0.55 |
| 10k200 | pGlu100 | 7.36 | 0.55 |
| 20k200 | pGlu100 | 8.48 | 0.55 |

## DLS/SAXS/TEM Concentrations

Table S3: Concentrations of charged monomers in DLS, SAXS, TEM experiments. The goal in these experiments was to measure the structure of charge-neutral PCMs.

| Neutral-Cationic Block Copolymer | Polyanion | Charged Monomer Concentration (mM) |
|---|---|---|
| 5k50 | pGlu50 | 0.90 |
| 10k50 | pGlu50 | 0.88 |
| 20k50 | pGlu50 | 0.88 |
| 5k100 | pGlu100 | 1.92 |
| 10k100 | pGlu100 | 1.77 |
| 20k100 | pGlu100 | 1.89 |
| 5k200 | pGlu200 | 1.85 |
| 10k200 | pGlu200 | 1.75 |
| 20k200 | pGlu200 | 2.02 |
| 5k50 | pGlu100 | 0.90 |
| 10k50 | pGlu100 | 0.88 |
| 20k50 | pGlu100 | 0.88 |
| 5k50 | pGlu200 | 1.79 |
| 10k50 | pGlu200 | 1.75 |
| 20k50 | pGlu200 | 1.75 |
| 5k100 | pGlu50 | 1.92 |
| 10k100 | pGlu50 | 1.77 |
| 20k100 | pGlu50 | 1.89 |
| 5k100 | pGlu200 | 1.92 |
| 10k100 | pGlu200 | 1.77 |
| 20k100 | pGlu200 | 1.89 |

| Neutral-Cationic Block Copolymer | Polyanion | Charged Monomer Concentration (mM) |
|---|---|---|
| 5k200 | pGlu50 | 1.85 |
| 10k200 | pGlu50 | 1.75 |
| 20k200 | pGlu50 | 2.02 |
| 5k200 | pGlu100 | 1.85 |
| 10k200 | pGlu100 | 1.75 |
| 20k200 | pGlu100 | 2.02 |

## Tabulated Results

## ITC Results

Table S4: ITC fits compiled for complexes formed from all block copolymer–homopolymer combinations and homopolymer–homopolymer combinations.

| Polycation | Polyanion | # of trials | Stoichiometry | ΔH (kJ/mol) | -TΔS (kJ/mol) |
|---|---|---|---|---|---|
| pLys30 | pGlu30 | 3 | 0.88 ± 0.06 | 1.07 ± 0.06 | −26.78 ± 0.34 |
| 5k30 | pGlu30 | 4 | 0.96 ± 0.15 | 1.18 ± 0.21 | −23.92 ± 0.26 |
| 10k30 | pGlu30 | 3 | 0.87 ± 0.03 | 1.09 ± 0.01 | −23.96 ± 0.14 |
| 20k30 | pGlu30 | 3 | 0.92 ± 0.06 | 1.20 ± 0.22 | −25.11 ± 0.61 |
| pLys50 | pGlu50 | 5 | 0.87 ± 0.10 | 1.65 ± 0.27 | −33.44 ± 0.34 |
| 5k50 | pGlu50 | 4 | 0.96 ± 0.06 | 1.47 ± 0.12 | −31.44 ± 0.40 |
| 10k50 | pGlu50 | 8 | 0.86 ± 0.09 | 1.56 ± 0.15 | −30.91 ± 0.57 |
| 20k50 | pGlu50 | 8 | 0.96 ± 0.06 | 1.27 ± 0.13 | −31.62 ± 0.36 |
| pLys100 | pGlu100 | 4 | 0.80 ± 0.04 | 1.58 ± 0.06 | −31.77 ± 0.42 |
| 5k100 | pGlu100 | 3 | 0.87 ± 0.07 | 1.60 ± 0.22 | −29.51 ± 0.50 |
| 10k100 | pGlu100 | 3 | 0.84 ± 0.10 | 1.60 ± 0.23 | −29.45 ± 0.44 |
| 20k100 | pGlu100 | 4 | 0.77 ± 0.09 | 1.67 ± 0.13 | −29.67 ± 0.14 |
| pLys250 | pGlu200 | 4 | 1.00 ± 0.04 | 1.77 ± 0.06 | −30.86 ± 0.64 |
| 5k200 | pGlu200 | 4 | 0.96 ± 0.10 | 1.58 ± 0.10 | −29.56 ± 0.27 |
| 10k200 | pGlu200 | 4 | 1.00 ± 0.06 | 1.43 ± 0.15 | −29.85 ± 0.46 |
| 20k200 | pGlu200 | 4 | 0.97 ± 0.04 | 1.99 ± 0.10 | −29.86 ± 0.57 |
| 5k50 | pGlu100 | 3 | 0.82 ± 0.06 | 1.35 ± 0.24 | −29.88 ± 0.87 |
| 10k50 | pGlu100 | 3 | 0.86 ± 0.04 | 1.59 ± 0.02 | −30.22 ± 1.19 |
| 20k50 | pGlu100 | 3 | 0.69 ± 0.05 | 1.69 ± 0.21 | −29.36 ± 0.62 |
| 5k50 | pGlu200 | 3 | 0.93 ± 0.02 | 1.57 ± 0.01 | −27.67 ± 0.07 |
| 10k50 | pGlu200 | 3 | 0.94 ± 0.04 | 1.50 ± 0.02 | −27.86 ± 0.26 |

| Polycation | Polyanion | # of trials | Stoichiometry | ΔH (kJ/mol) | -TΔS (kJ/mol) |
|---|---|---|---|---|---|
| 20k50 | pGlu200 | 3 | 0.99 ± 0.03 | 1.31 ± 0.31 | −27.71 ± 0.85 |
| 5k100 | pGlu50 | 3 | 0.84 ± 0.07 | 1.53 ± 0.05 | −31.57 ± 0.35 |
| 10k100 | pGlu50 | 3 | 0.90 ± 0.01 | 1.4 ± 0.06 | −31.95 ± 0.23 |
| 20k100 | pGlu50 | 3 | 0.88 ± 0.06 | 1.56 ± 0.22 | −31.10 ± 0.38 |
| 5k100 | pGlu200 | 3 | 0.90 ± 0.01 | 1.64 ± 0.03 | −29.37 ± 0.40 |
| 10k100 | pGlu200 | 3 | 0.97 ± 0.06 | 1.45 ± 0.15 | −29.33 ± 0.37 |
| 20k100 | pGlu200 | 3 | 0.88 ± 0.01 | 1.61 ± 0.24 | −29.15 ± 0.32 |
| 5k200 | pGlu50 | 3 | 0.80 ± 0.04 | 1.67 ± 0.11 | −32.46 ± 0.87 |
| 10k200 | pGlu50 | 3 | 0.84 ± 0.06 | 1.56 ± 0.09 | −32.56 ± 0.16 |
| 20k200 | pGlu50 | 3 | 0.76 ± 0.06 | 1.90 ± 0.02 | −32.11 ± 0.10 |
| 5k200 | pGlu100 | 3 | 0.72 ± 0.02 | 1.60 ± 0.07 | −30.53 ± 0.42 |
| 10k200 | pGlu100 | 3 | 0.75 ± 0.05 | 1.46 ± 0.03 | −30.62 ± 0.16 |
| 20k200 | pGlu100 | 3 | 0.67 ± 0.03 | 1.89 ± 0.06 | −30.76 ± 0.29 |

## Statistical Comparison of Entropy of Complexation (ANOVA Tests)

| | 5k30 | 10k30 | 20k30 |
|---|---|---|---|
| **pLys30** | <0.0001 | <0.0001 | 0.0017 |
| **5k30** | | 0.999 | 0.0097 |
| **10k30** | | | 0.0174 |

| | 5k50 | 10k50 | 20k50 |
|---|---|---|---|
| **pLys50** | <0.0001 | <0.0001 | <0.0001 |
| **5k50** | | 0.2458 | 0.9143 |
| **10k50** | | | 0.0225 |

| | 5k100 | 10k100 | 20k100 |
|---|---|---|---|
| **pLys100** | <0.0001 | <0.0001 | <0.0001 |
| **5k100** | | 0.9962 | 0.9508 |
| **10k100** | | | 0.8719 |

| | 5k200 | 10k200 | 20k200 |
|---|---|---|---|
| **pLys250/ pGlu200** | 0.0157 | 0.0655 | 0.0681 |
| **5k200** | | 0.8442 | 0.8334 |
| **10k200** | | | >0.9999 |

Figure S2: P-values for ANOVA tests comparing BCP–HP and HP–HP complexation for matching charged block lengths. The p-values in red show the statistical significance between HP–HP complexation and BCP–HP complexation directly, highlighting the importance of the presence of the PEG block. The lack of statistical significance in the other p-values shows that the length of the PEG block does not affect the entropy of complexation.

## DLS and SAXS Results

Table S5: DLS and SAXS fits compiled for PCMs formed from all block copolymer–homopolymer combinations.

| PCM Components | | SAXS | | | | DLS | | |
|---|---|---|---|---|---|---|---|---|
| Block Copolymer | Polyanion | $I_0$ ($cm^{-1}$) | $R_{core}$ (nm) | Aspect Ratio | PDI | $R_h$ (nm) | SD (nm) | Corona Thickness (nm) |
| 5k50 | pGlu50 | 0.70 | 12.9 | 1.6 | 0.01 | 28.4 | 2.43 | 15.5 |
| 10k50 | pGlu50 | 0.58 | 11.0 | 2.0 | 0.01 | 33.6 | 4.7 | 22.6 |
| 20k50 | pGlu50 | 1.25 | 10.0 | 2.0 | 0.06 | 37.2 | 5.63 | 27.2 |
| 5k100 | pGlu100 | 4.64 | 20.5 | 1.5 | 0.02 | 33.4 | 1.86 | 12.9 |
| 10k100 | pGlu100 | 2.06 | 15.1 | 1.7 | 0.01 | 31.8 | 1.62 | 16.8 |
| 20k100 | pGlu100 | 1.81 | 14.2 | 1.9 | 0.02 | 33.4 | 3.22 | 19.2 |
| 5k200 | pGlu200 | 11.05 | 27.2 | 1.0 | 0.02 | 41.0 | 0.72 | 13.8 |
| 10k200 | pGlu200 | 8.90 | 25.5 | 1.1 | 0.04 | 46.6 | 1.04 | 21.1 |
| 20k200 | pGlu200 | 4.68 | 18.0 | 1.7 | 0.01 | 39.9 | 0.51 | 21.9 |
| 5k50 | pGlu100 | 0.44 | 12.9 | 1.5 | 0.01 | 23.6 | 1.10 | 10.7 |
| 10k50 | pGlu100 | 0.31 | 11.6 | 1.7 | 0.02 | 24.6 | 1.07 | 13.0 |
| 20k50 | pGlu100 | 0.34 | 10.1 | 2.0 | 0.06 | 27.0 | 1.05 | 17.0 |
| 5k50 | pGlu200 | 0.62 | 13.2 | 1.1 | 0.03 | 20.8 | 0.42 | 7.6 |
| 10k50 | pGlu200 | 0.56 | 11.1 | 1.3 | 0.05 | 24.3 | 1.01 | 13.3 |
| 20k50 | pGlu200 | 0.51 | 9.6.0 | 1.7 | 0.09 | 27.2 | 0.72 | 17.6 |
| 5k100 | pGlu50 | 4.84 | 20.8 | 1.4 | 0.03 | 34.1 | 0.86 | 13.3 |
| 10k100 | pGlu50 | 2.08 | 14.8 | 1.8 | 0.01 | 30.3 | 1.72 | 15.5 |
| 20k100 | pGlu50 | 2.02 | 14.1 | 2.0 | 0.01 | 33.0 | 1.90 | 18.8 |
| 5k100 | pGlu200 | 3.68 | 20.7 | 1.4 | 0.25 | 34.0 | 0.83 | 13.2 |
| 10k100 | pGlu200 | 1.63 | 15.0 | 1.7 | 0.01 | 31.3 | 0.73 | 16.3 |

| PCM Components | | SAXS | | | | DLS | | |
|---|---|---|---|---|---|---|---|---|
| Block Copolymer | Polyanion | $I_0$ ($cm^{-1}$) | $R_{core}$ (nm) | Aspect Ratio | PDI | $R_h$ (nm) | SD (nm) | Corona Thickness (nm) |
| 20k100 | pGlu200 | 1.44 | 14.1 | 1.9 | 0.02 | 35.8 | 0.83 | 21.8 |
| 5k200 | pGlu50 | 5.15 | 29.2 | 1.2 | 0.03 | 45.0 | 1.17 | 15.8 |
| 10k200 | pGlu50 | 11.84 | 27.4 | 1.0 | 0.09 | 47.2 | 1.20 | 19.8 |
| 20k200 | pGlu50 | 3.51 | 21.1 | 1.6 | 0.01 | 43.2 | 1.54 | 22.1 |
| 5k200 | pGlu100 | 14.82 | 30.0 | 1.7 | 0.01 | 44.6 | 0.99 | 14.6 |
| 10k200 | pGlu100 | 8.81 | 29.1 | 1.0 | 0.04 | 47.6 | 1.52 | 18.6 |
| 20k200 | pGlu100 | 4.40 | 20.2 | 1.6 | 0.02 | 40.6 | 0.71 | 20.4 |

## MALS Data for Select Formulations

Table S6: Number of BCP chains per micelle $n_{BCP}$ for select combinations of block lengths as measured by MALS. Taking the MALS data and performing a Zimm analysis, the weight-averaged molecular weight is calculated by extrapolating to zero angle and zero concentration. $n_{BCP}$ is calculated by assuming charge neutrality of PCMs.

| pLys Block Length | PEG Block MW (kDa) | pGlu50 | pGlu100 | pGlu200 |
|---|---|---|---|---|
| 50 | 5 | 94 | 126 | - |
| | 10 | 42 | 91 | 62 |
| | 20 | 26 | 25 | 34 |
| 100 | 5 | - | 195 | 316 |
| | 10 | - | 79 | 96 |
| | 20 | 64 | 41 | 64 |
| 200 | 5 | - | 316 | - |
| | 10 | - | 354 | 164 |
| | 20 | 163 | 108 | 80 |

## CBQCA Data for Select Formulations

Table S7: Percent of BCP incorporated into PCMs for various BCP-anion combinations as measured by a CBQCA assay. DLS measurements of both the filtrate and retentate were taken to ensure only the retentate contained micelles for all samples. Most PCM formulations show that the vast majority of BCPs are found in PCMs within the retentate. As a further test, PCM formulations containing twice as many cation monomers (in the BCP) compared to anion monomers (in the homopolyanion) were also tested with the same assay, for which the percentages of BCPs present in the PCM were well below 100%. Ideally, for a 2:1 ratio of cation:anion, 50% of BCPs would be present in the PCM while the remainder would be free within solution.

| BCP | Anion | % BCP in PCM |
|---|---|---|
| 5k50 | pGlu50 | ~100 |
| 10k50 | pGlu50 | ~100 |
| 20k50 | pGlu50 | 94.7 |
| 5k100 | pGlu100 | 86.3 |
| 10k100 | pGlu100 | ~100 |
| 5k100 | pGlu50 | ~100 |
| 10k100 | pGlu50 | ~100 |
| 5k100 | pGlu50 | ~100 |
| 5k100 (2:1 cation-anion ratio) | pGlu100 | 34.0 |
| 10k100 (2:1 cation-anion ratio) | pGlu100 | 50.7 |

## Scattering Length Density (SLD) Calculation

Table S8: Scattering Length Density (SLD) and contrast squared values of PCM components relative to the solvent (water/PBS).

| **Material** | **SLD ($\rho$) ($\times 10^{10}$ cm$^{-2}$)** | **Contrast$^2$ ($\Delta\rho$)$^2$ ($\times 10^{20}$ cm$^{-4}$)** |
|---|---|---|
| Lysine | 12.94 | 12.0 |
| Glutamic Acid | 12.08 | 6.8 |
| PEG | 10.36 | 0.8 |
| Solvent | 9.47 | 0 |

The SLD of the PCM charged core is taken by calculating the volume-fraction-averaged SLD of the individual components, namely water, lysine, and glutamic acid. It is assumed that there are an equal number of lysine and glutamic acid monomers in each PCM core (one ion pair consists of one of each monomer), so the volume fraction of each monomer corresponds to the volume of each monomer (170 ˚A$^3$ for Lys and 141 ˚A$^3$ for Glu).[1] The PCM core is also significantly hydrated, with water volume fraction that is assumed to be similar to that of the analogous homopolyelectrolyte coacervate system. In the case of the pLys/pGlu system, this has previously been measured to be $\phi_{H2O} = 0.26$.[2] With these considerations taken into account, $\Delta\rho_{core} = 6.4 \times 10^{19}$ cm$^{-4}$.

## Comparing PCM Aggregation Numbers Calculated from MALS and SAXS

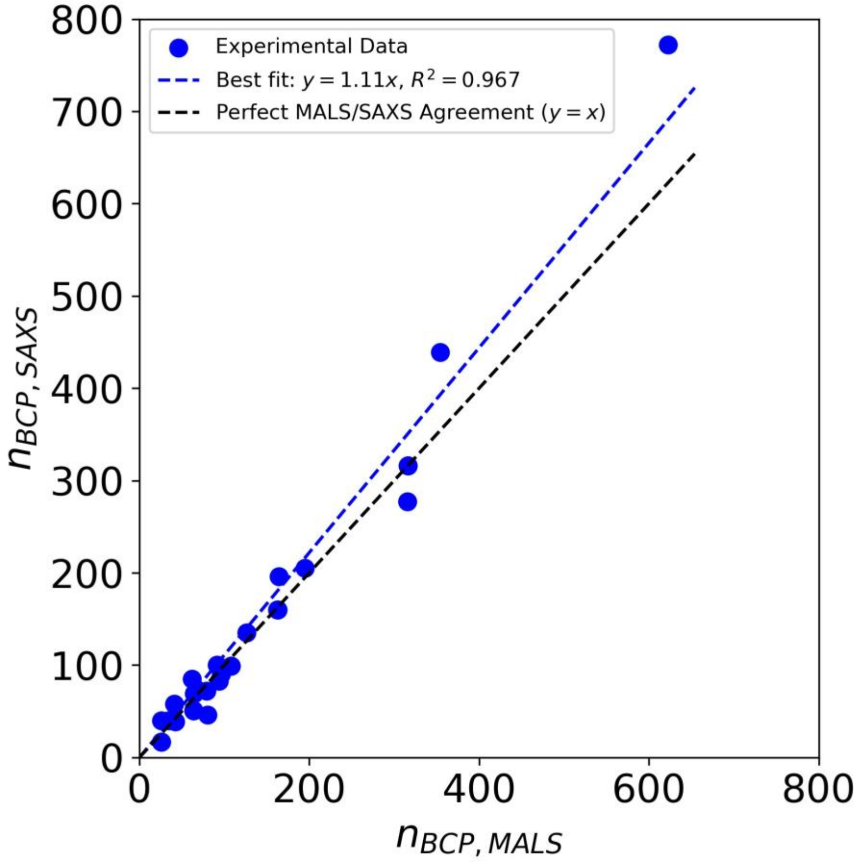


Figure S3: $n_{BCP}$ as calculated by SAXS (Table 6) and MALS (Table S6) show agreement across PCM formulations, supporting the SAXS analysis performed that assumes free polymer concentration is negligible compared to polymer concentration present in a PCM.

**Schematic of Hypothesis Regarding PEG Chain Conformation and Complexation Entropy**

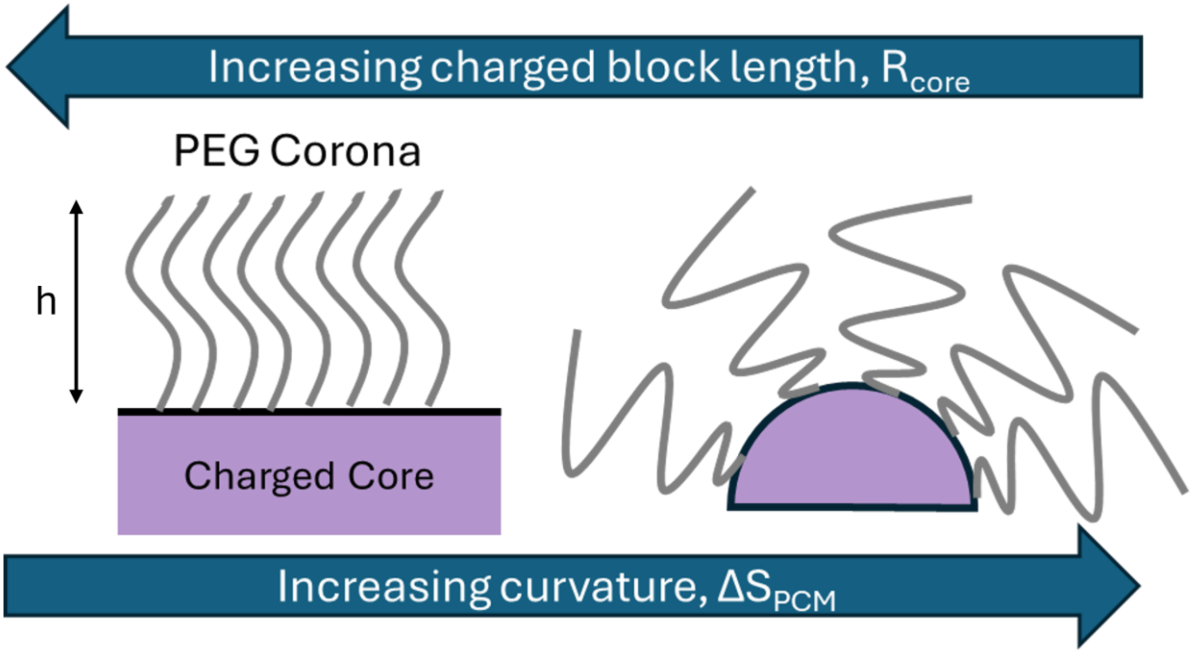


Figure S4: Schematic summarizing findings of self-consistent field theory[3] (SCFT) as they relate to the conformation of chains tethered to a curved interface. When applied to PCMs, as the curvature of the PCM core-corona interface increases for smaller PCMs, there is more room for corona PEG chains to splay, leading to a decreased brush height *h*. Thermodynamically, this would imply that PCMs with a smaller core would yield entropically more favorable corona chains (greater $\Delta S$) due to the core curvature effect.

## PCM Structure and Entropy of Complexation

## Mismatched Charged Block Length

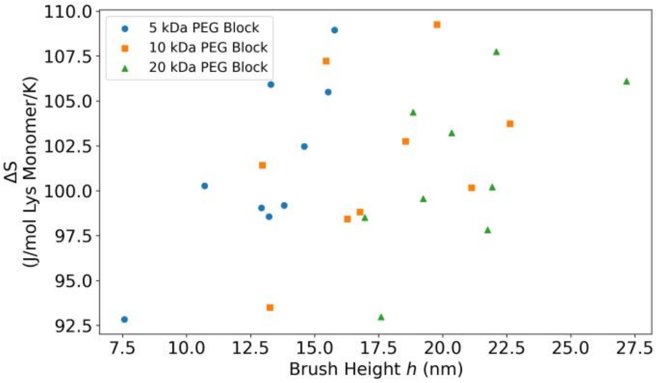


(a)

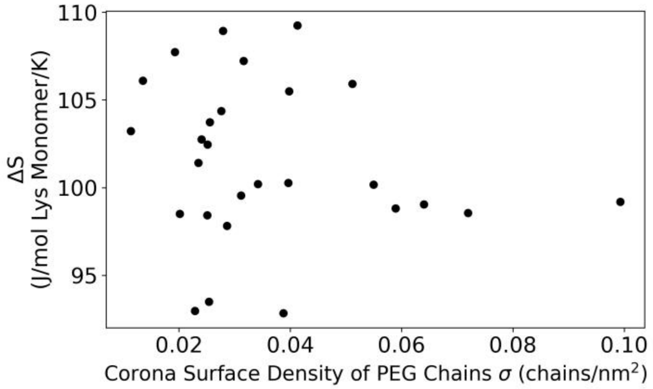


(b)

Figure S5: (a) Entropy of complexation as a function of brush height for mismatched charged block length PCMs. Data are segregated for different corona PEG block lengths. We see that within each group, there is a slight increase in entropy of complexation as the brush height increases. If the entropy of complexation were dependent on the stretching of the corona PEG chains, there would be a decrease in entropy with increasing brush height, which is not the case here. (b) For the same set of PCMs, we see no dependence of the entropy of complexation on the corona chain surface density $\sigma$, which also suggests that the entropy is not based strictly on the packing of the PEG chains on the surface.

## Matched Charged Block Length

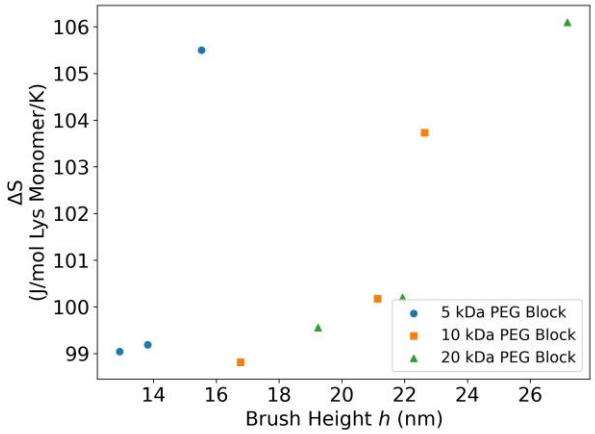


(a)

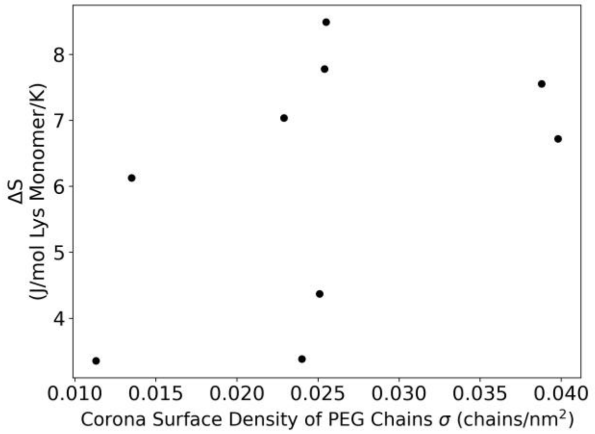


(b)

Figure S6: (a) Entropy of complexation as a function of brush height for matched charged block length PCMs. Data are segregated for different corona PEG block lengths. We see that within each group, a larger brush height leads to an increase in the entropy of complexation, which runs counter to the hypothesis that the extended nature of PEG chains plays a role in PCM formation. (b) For the same set of PCMs, we see no dependence of the entropy of complexation on the corona surface density $\sigma$.

### Reduced Tethering Density of PCM Formulations

Table S9: Reduced tethering density ($\tilde{\sigma}$) for all combinations of block lengths that led to monodisperse PCM formation. This nondimensionalized metric for chain crowding at an interface can be thought of as the number of tethered chains within the same area as a chain in an unperturbed conformation in the same solvent. Values exceeding 3 have been shown to induce chain squeezing resulting in the swelling described in Figure S4.[4,5] However, for these formulations, the majority of values are below this threshold, where the chains don't feel each other's presence. This may explain why the entropy of complexation is independent of PEG block length.

| pLys Block Length | PEG Block MW (kDa) | pGlu50 | pGlu100 | pGlu200 |
|---|---|---|---|---|
| 50 | 5 | 0.99 | 1.59 | 2.47 |
| | 10 | 1.42 | 3.28 | 3.06 |
| | 20 | 1.68 | 3.87 | 4.25 |
| 100 | 5 | 0.99 | 0.97 | 1.27 |
| | 10 | 1.31 | 1.41 | 1.76 |
| | 20 | 2.50 | 2.85 | 3.43 |
| 200 | 5 | 1.79 | 0.70 | 0.63 |
| | 10 | 1.39 | 2.30 | 1.34 |
| | 20 | 3.56 | 2.40 | 1.41 |

## References


(1) Harpaz, Y.; Gerstein, M.; Chothia, C. Volume Changes on Protein Folding. *Structure* **1994**, *2*, 641–649.

(2) Li, L.; Srivastava, S.; Andreev, M.; Marciel, A. B.; de Pablo, J. J.; Tirrell, M. V. Phase Behavior and Salt Partitioning in Polyelectrolyte Complex Coacervates. *Macromolecules* **2018**, *51*, 2988–2995.

(3) Dan, N.; Tirrell, M. Polymers Tethered to Curves Interfaces: A Self-Consistent-Field Analysis. *Macromolecules* **1992**, *25*, 2890–2895.

(4) Tockary, T. A.; Osada, K.; Chen, Q.; Machitani, K.; Dirisala, A.; Uchida, S.; Nomoto, T.; Toh, K.; Matsumoto, Y.; Itaka, K.; Nitta, K.; Nagayama, K.; Kataoka, K. Tethered PEG

Crowdedness Determining Shape and Blood Circulation Profile of Polyplex Micelle Gene Carriers. *Macromolecules* **2013**, *46*, 6585–6592.

(5) Chen, W. Y.; Zheng, J. X.; Cheng, S. Z. D.; Li, C. Y.; Huang, P.; Zhu, L.; Xiong, H.; Ge, Q.; Guo, Y.; Quirk, R. P.; Lotz, B.; Deng, L.; Wu, C.; Thomas, E. L. Onset of Tethered Chain Overcrowding. *Physical Review Letters* **2004**, *93*.
